# Photocatalytic Hydrogen Production using Polymeric Carbon Nitride with a Hydrogenase and a Bioinspired Synthetic Ni Catalyst**

**DOI:** 10.1002/anie.201406811

**Published:** 2014-09-09

**Authors:** Christine A Caputo, Manuela A Gross, Vincent W Lau, Christine Cavazza, Bettina V Lotsch, Erwin Reisner

**Affiliations:** Christian Doppler Laboratory for Sustainable SynGas Chemistry, Department of Chemistry, University of CambridgeLensfield Road, Cambridge CB2 1EW (UK); Max Planck Institute for Solid State Research, Department of Chemistry, Ludwig-Maximilians-Universität MünchenHeisenbergstrasse 1, 70569 Stuttgart (Germany), Butenandtstrasse 5–13 (Haus D), 81377 München (Germany); CEA, CNRS, Université Grenoble Alpes, IBS71 Avenue des Martyrs, 38044 Grenoble (France)

**Keywords:** carbon nitride, enzyme catalysis, hydrogen production, hydrogenases, photocatalysis

## Abstract

Solar-light-driven H_2_ production in water with a [NiFeSe]-hydrogenase (H_2_ase) and a bioinspired synthetic nickel catalyst (NiP) in combination with a heptazine carbon nitride polymer, melon (CN_x_), is reported. The semibiological and purely synthetic systems show catalytic activity during solar light irradiation with turnover numbers (TONs) of more than 50 000 mol H_2_ (mol H_2_ase)^−1^ and approximately 155 mol H_2_ (mol NiP)^−1^ in redox-mediator-free aqueous solution at pH 6 and 4.5, respectively. Both systems maintained a reduced photoactivity under UV-free solar light irradiation (λ>420 nm).

Efficient and noble metal-free water photolysis using sunlight is a primary focus of research to advance sustainable solar energy generation.[1] Photocatalytic H_2_ production can be achieved by employing hybrid systems with a solid-state light absorber such as an inorganic semiconductor assisted by a metallic, synthetic, or enzymatic electrocatalyst.[2] These systems typically contain expensive, inefficient, and/or unstable components, but high performance solar fuel devices need to be constructed from parts without these limitations.

Hydrogenases (H_2_ases) are H_2_-cycling enzymes and are by far the most efficient noble-metal-free electrocatalysts for H_2_ generation with an unrivalled turnover frequency (TOF) benchmark of more than 10^3^ s^−1^ even at a modest overpotential.[3] This excellent electrocatalytic activity of H_2_ases was exploited in photocatalytic schemes with a light absorber in the absence of a soluble redox mediator: a homogeneous photocatalytic system with a molecular organic dye,[4] and semiheterogeneous systems, in which the H_2_ase is immobilized on Ru dye-sensitized TiO_2_ nanoparticles,[5] and on Cd-containing quantum dots,[6] displaying excellent photocatalytic activity in sacrificial schemes.

An efficient class of H_2_ase-inspired synthetic catalysts containing non-noble metal centers have been developed by DuBois and co-workers.[7] They possess a Ni bis(diphosphine) ligand core bearing pendant amino groups, which, much like those found in the active site of [FeFe]-H_2_ases,[8] can act as catalytically active proton relays in the second coordination sphere of the 3d metal center. Photocatalytic H_2_ generation with such Ni bis(diphosphine) catalysts has only been achieved in combination with a costly Ru dye in purely aqueous solution.[9]

Amorphous polymeric carbon nitride (CN_*x*_) with a poly(tri-*s*-triazine) (polyheptazine) building block (often referred to as melon or g-C_3_N_4_) has recently emerged as an attractive visible-light absorber and can generate H_2_ photocatalytically.[10] It can be easily synthesized by condensation of cyanamide, dicyandiamide, or melamine at elevated temperatures and displays high activities and photostability of more than 72 h.[10b] The material has well-suited band positions for water splitting and a band gap of approximately 2.7 eV with a conduction band potential at −0.8 V vs. RHE.[10a,b] Co-catalyst integration of non-noble metals,[11] Pt,[10b], [12] Ni(TEOA)_3_^2+^ (TEOA=triethanolamine),[13] and cobaloximes[14] with CN_*x*_ has previously been used as a strategy to enhance H_2_ evolution rates.

In this study, we report a photocatalytic CN_*x*_–enzyme hybrid system for visible-light-driven H_2_ generation (Figure 1). This CN_*x*_–H_2_ase hybrid assembly operates in an aqueous sacrificial electron donor solution and does not require an expensive or fragile light absorber for visible light promoted photocatalysis as reported for the previous H_2_ase-based systems. We selected *Desulfomicrobium baculatum* (*Dmb*) [NiFeSe]-H_2_ase because of its well-known[15] and excellent H_2_ evolution rate as well as tolerance toward H_2_ and O_2_, allowing for the accumulation of H_2_ during irradiation and the handling of the enzyme in the absence of strictly anaerobic conditions.[4], [5], [15b]

**Figure 1:**
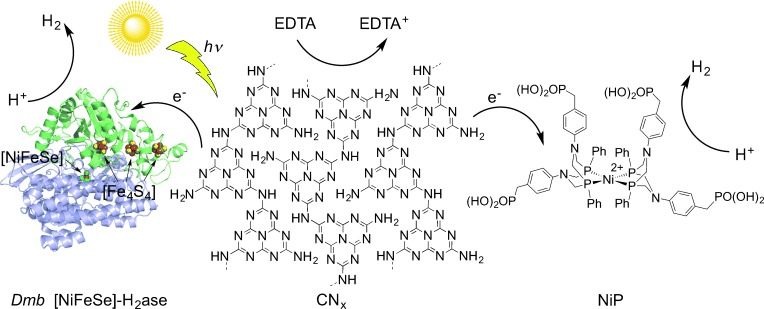
Representation of the photo-H_2_ production with CN_*x*_ and *Dmb* [NiFeSe]-H_2_ase (PDB ID: 1CC1)[15a] or CN_*x*_ and NiP (counterions omitted) in aqueous EDTA solution. Irradiation of CN_*x*_ results in the photoinduced direct electron transfer to the catalysts with H_2_ formation and hole quenching in CN_*x*_ by EDTA.

The photocatalytic H_2_ generation systems were assembled in a photoreactor (total volume 7.74 mL) by dispersing CN_*x*_ (5 mg, approx. 1 μm-sized particles with a Brunauer–Emmett–Teller surface area of 9 m^2^ g^−1^; see Figures S1–S4 for FTIR, XRD, SEM, and zeta-potential measurements) in an aqueous solution of the electron donor (0.1 m, 3 mL). The catalyst (H_2_ase or NiP, see below) was added to the suspension and the light-protected reactor was sealed and purged with 2 % CH_4_ (as an internal gas chromatography standard) in N_2_ before irradiating the stirred mixture at 25 °C. Irradiation was provided by a solar light simulator (air mass 1.5 G, 100 mW cm^−2^) and headspace H_2_ was quantified at regular time intervals by gas chromatography. The reaction conditions were optimized for a high rate of H_2_ production per catalyst (as expressed by the TOF) by varying the pH of the solution, the amount of catalyst and by screening different electron donors (Table S1; Figures S5 and S6).

The optimized standard system for CN_*x*_–H_2_ase comprises 50 pmol H_2_ase with 5 mg melon in 3 mL ethylenediamine tetraacetic acid (EDTA, 0.1 m) at pH 6 under simulated solar irradiation at *λ*>300 nm (Figure 2). Under these conditions, a TOF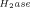 of (5532±553) mol H_2_ (mol H_2_ase)^−1^ h^−1^ and (55.3±5.5) μmol H_2_ (g CN_*x*_)^−1^ h^−1^ are photogenerated with almost linear H_2_ evolution, producing (0.82±0.08) μmol H_2_ during the first four hours. Photoinduced direct electron transfer from CN_*x*_ to the H_2_ase was therefore observed, making a soluble redox mediator unnecessary. The CN_*x*_–H_2_ase suspension was photoactive for 48 h, whereupon (2.5±0.2) μmol of H_2_ was produced with a TON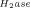 of >50 000. Control experiments in the dark and in the absence of CN_*x*_ or H_2_ase showed no H_2_ formation. Only minimal amounts of H_2_ were produced when the electron donor buffer EDTA was replaced by potassium phosphate buffer (KP_i_; 41 mm, pH 7, Table S1).

**Figure 2:**
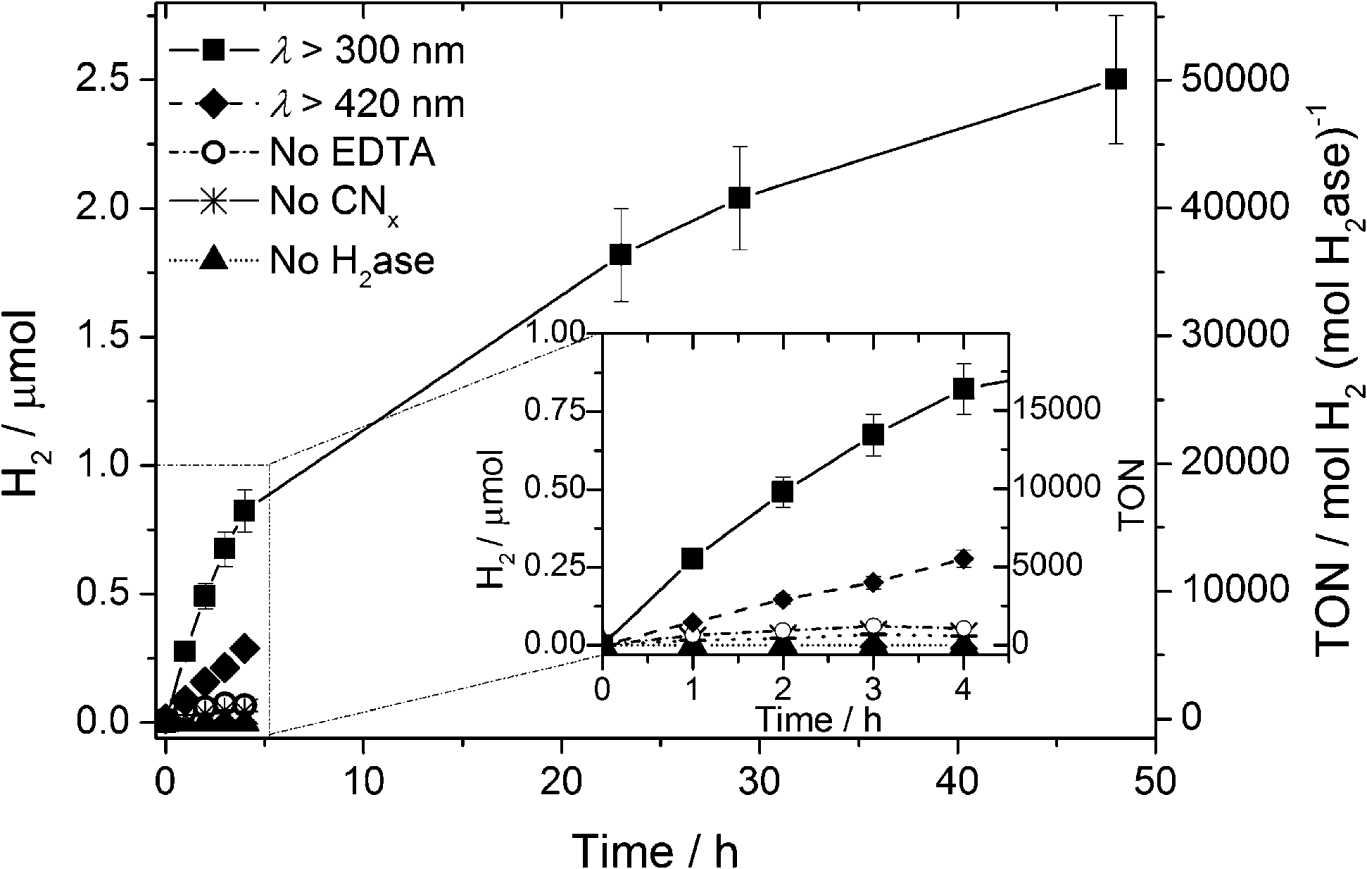
H_2_ production under optimized conditions using *Dmb* [NiFeSe]-H_2_ase (50 pmol) in EDTA (pH 6, 0.1 m, 3 mL) and CN_*x*_ (5 mg) under 1 sun irradiation in the absence (*λ*>300 nm) and presence of a 420 nm UV filter. Control experiments without EDTA, CN_*x*_, or H_2_ase are also shown.

The amount of H_2_ase per mg of CN_*x*_ and the light intensity were varied to gain insight into the performance-limiting factors of the CN_*x*_–H_2_ase hybrid. Increasing the H_2_ase loading from 50 to 200 pmol per 5 mg CN_*x*_ resulted in a linear increase in overall H_2_ generation with an unchanged TOF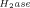 (Table S1, Figure S6). Decreasing the solar light intensity with neutral density filters from 100 to 50 mW cm^−2^ did not result in a significant reduction of the photoactivity, although a further reduction to 20 mW cm^−2^ resulted in approximately 40 % decreased activity (Table S2; Figure S7). These experiments suggest that the optimized CN_*x*_–H_2_ase system is not limited by light absorption at CN_*x*_, and support that enzyme adsorption and interaction with the CN_*x*_ is performance limiting (see below).

The CN_*x*_–H_2_ase system was also studied under visible light irradiation (λ>420 nm). A decrease in photoactivity was observed giving rise to a TOF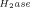 of (768±77) h^−1^, which corresponds to 14 % of the activity under UV/Vis irradiation (Figure 2). This can be attributed to the significantly reduced light absorption of CN_*x*_ above 420 nm (Figure S8). The external quantum efficiency (EQE) of the system was determined by irradiation of samples under standard conditions using a monochromatic LED light source at two wavelengths (*λ*=365 nm, *I*=3.5 mW cm^−2^ and *λ*=460 nm, *I*=47 mW cm^−2^). UV-irradiation gave an unoptimized EQE of approximately 7×10^−2^ %, whereas an EQE of 5×10^−3^ % was obtained at *λ*=465 nm (Figure S8).

A centrifugation test was performed to gain insight into the strength of interaction between the enzyme and CN_*x*_ particles. First, H_2_ production was monitored for 2 h with CN_*x*_–H_2_ase under standard conditions. The suspension was then centrifuged (5000 rpm, 5 min) followed by washing the pellet with water and redispersion of the particles in aqueous EDTA (0.1 m, pH 6). This suspension was then irradiated again after purging the headspace with 2 % CH_4_ in N_2_. The remaining activity of this mixture was 12 % relative to the activity prior to centrifugation, indicating that a relatively weak interaction suffices for electron transfer to occur from CN_*x*_ to the H_2_ase. Physical adsorption of the H_2_ase on the CN_*x*_ surface can be expected and we speculate that the H_2_ase[16] may form hydrogen bonds with the =NH=, terminal =NH_2_ or Lewis basic heptazine edge nitrogens in CN_*x*_.[10a], [17] The isoelectric point of CN_*x*_ was determined by zeta potential measurements as 3.3[18] and, at pH 6, the surface of CN_*x*_ is therefore negatively charged (≈−15 mV) (Figure S4).

Although the direct electron transfer was observed from the photoexcited CN_*x*_ to the H_2_ase, the CN_*x*_–H_2_ase system displayed a significantly increased photoactivity under standard conditions upon addition of an excess of the redox mediator, methyl viologen (MV),[19] producing up to 18.7 μmol H_2_ after 4 h (Figure S9). A long-term experiment with H_2_ase (50 pmol), CN_*x*_ (5 mg), and added MV (5 μmol) in aqueous EDTA (0.1 m) at pH 6 was also performed. The photoreactor was purged with 2 % CH_4_/N_2_ after 24 and 48 h and additional MV (5 μmol) was added at the same time intervals. After 69 h, the CN_*x*_–MV–H_2_ase system produced 77 μmol H_2_ with a TON of 1.5×10^6^ and an initial TOF of 12.3 s^−1^ (Figure S10). Replenishment of MV was required due to decomposition of the organic mediator during irradiation. The substantially increased H_2_ production activity in the presence of MV suggests that the electron transfer from CN_*x*_ to H_2_ase is not yet fully optimized, presumably due to weak and nonspecific interactions at the CN_*x*_–H_2_ase interface.

Steady-state photoluminescence (PL) measurements were also performed with the CN_*x*_ in suspension upon photoexcitation at *λ*=365 nm and following the PL emission at 450 nm (Figure S11). The PL emission of sonicated CN_*x*_ (0.22 g mL^−1^ in 0.1 m EDTA pH 6) is more strongly quenched upon addition of 50 pmol MV compared to 50 pmol H_2_ase. These results further support that the photoinduced electron transfer from CN_*x*_ to MV is more efficient than that to the H_2_ase.

The reported semibiological hybrid system provides a novel “per active site” activity benchmark for a cocatalyst on a CN_*x*_ material.[7g], [11a,b], [20] Photocatalytic H_2_ generation schemes previously reported with H_2_ases and other light absorbers show a high TOF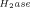 (approximately 10^6^ h^−1^), but these systems rely on an expensive (Ru dye), toxic (Cd-based quantum dot), and/or fragile (organic dye) visible light absorber.[4], [5b], [6c] This study demonstrates that the biocompatibility of CN_*x*_ can be exploited to overcome these limitations and that by improving the coupling of CN_*x*_ to the H_2_ase, the photoactivity will be further enhanced.

Successful H_2_ production with CN_*x*_–H_2_ase prompted us to investigate a water-soluble and functional synthetic H_2_ase-mimic, [Ni^II^(P^Ph^_2_{NPhCH_2_P(O)(OH)_2_}_2_)_2_]Br_2_ (NiP; Figure 1),[9] for comparison. Ni bis(diphosphine)[7a–e] complexes are among the most active H_2_ generation electrocatalysts and, importantly, NiP has recently been shown to act as an excellent electrocatalyst in aqueous solution.[9] The purely synthetic CN_*x*_–NiP assembly is photoactive and conditions were optimized for the highest TOF_NiP_. Aqueous EDTA solutions (0.1 m) at pH 4.5 containing NiP (20 nmol) and suspended CN_*x*_ (5 mg) were studied under simulated solar irradiation at *λ*>300 nm (Table S3, Figures S12–S14). Under these conditions, solar H_2_ generation by CN_*x*_–NiP gave an initial activity of (437.1±43.7) μmol H_2_ (g CN_*x*_)^−1^ h^−1^ producing (2.2±0.2) μmol H_2_ in the first hour and giving a TOF_NiP_ of (109.3±10.9) mol H_2_ (mol NiP)^−1^ h^−1^. CN_*x*_-NiP was photoactive for three hours, whereupon (3.3±0.4) μmol of H_2_ with a TON of (166.1±20.6) mol H_2_ (mol NiP)^−1^ was produced (Figure 3). A 64 % decrease in photocatalytic H_2_ generation yield was observed for CN_*x*_–NiP when irradiating with *λ*>420 nm instead of >300 nm solar light. Decomposition of NiP is the likely reason for the ceased activity after three hours, because the photoactivity is fully regenerated if additional NiP is added (Figure S15).

**Figure 3:**
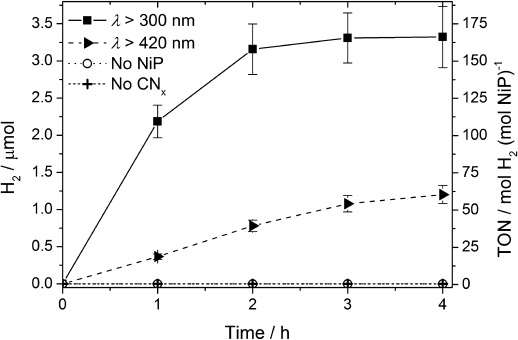
H_2_ production under optimized conditions using NiP (20 nmol) in aqueous EDTA (0.1 m, pH 4.5, 3 mL) and CN_*x*_ (5 mg) under 1 sun irradiation. Data collected under standard conditions (*λ*>300 nm), with UV-light-filtered irradiation (*λ*>420 nm) and control experiments without NiP catalyst or CN_*x*_ are also shown.

Photo-H_2_ generation with CN_*x*_–NiP is thus significantly higher than for previously reported CN_*x*_ systems with immobilized noble-metal-free cocatalysts in aqueous solution. A TOF of <0.5 h^−1^ and a TON of 4 was reported for a cobaloxime, [CoCl(dimethylglyoximato)_2_(pyridine)], after 8 h irradiation with CN_*x*_ in aqueous TEOA at pH 10.4.[14a] Other systems comprising a cobaloxime with a pyrene-functionalized pyridine[14c] and NiCl_2_ with TEOA and CN_*x*_[13] showed TONs of 160 and 281 and TOFs of approximately 40 and 6.7 h^−1^, respectively, but required excess organic solvent. Previously, photo-H_2_ generation with NiP was only reported with a molecular Ru dye, in which a TOF_NiP_ of up to 460 h^−1^ and a TON_NiP_ of up to 723 in pH 4.5 ascorbic acid solution were reported.[9]

The photo-H_2_ generation activity of CN_*x*_–NiP is dependent on the NiP concentration (Figures S13 and S14) and reduction of the light intensity (*I*) with neutral density filters has a substantial impact on the photoactivity. The NiP-based TOF decreases from (71.1±7.1) h^−1^ (*I*=100 %) to (32.4±3.2) (*I*=50 %) and (13.1±1.4) h^−1^ (*I*=20 %; Table S4; Figure S16). The purely synthetic system is therefore limited both by catalyst concentration and light absorption. The unoptimized EQE for the CN_*x*_–NiP system was determined to be (0.37±0.02) % under UV light (*λ*=365 nm) and (0.04±0.01) % under blue light irradiation (*λ*=460 nm) after 2 h. The wavelength-dependent EQE is consistent with the decrease in light absorption by CN_*x*_ at higher wavelengths (Figure S17).

Centrifugation experiments in analogy to the enzyme system were performed to examine the strength of the interaction between CN_*x*_ and NiP. After centrifugation, washing, and redispersion in fresh EDTA solution, 8 % of the photoactivity remained for the synthetic system implying a weak interaction between the CN_*x*_ and NiP (Figure S18). Electronic absorption spectrophotometry was used to quantify the amount of NiP adsorbed to CN_*x*_. By comparing UV-visible spectra of NiP (6.7 μm; *λ*_max_=320 and 450 nm) in aqueous EDTA solution (3 mL; 0.1 m, pH 4.5) before and after the addition of CN_*x*_ and centrifugation, an estimate of approximately 20 % NiP was adsorbed on CN_*x*_ (Figure S19). The physical adsorption and H-bonding between the phosphonic acid groups in NiP and the terminal =NH_2_ and =NH= groups in CN_*x*_ are possible modes of interaction.[10a], [17]

The addition of MV (20 μmol) to a standard photocatalytic experiment showed an approximately 20 % decreased H_2_ production activity. The reaction mixture turned dark blue upon irradiation, indicative of the presence of reduced MV, and implies that MV successfully scavenged electrons from the photoexcited CN_*x*_, but was unable to transfer them to NiP (Figure S20).

The comparison of the CN_*x*_–H_2_ase with the CN_*x*_–NiP hybrid system shows the expected higher “per active site” activity of the enzymatic system, whereas the purely synthetic system shows an overall higher H_2_ production rate due to the larger amount of NiP (20 nmol) used compared to H_2_ase (50 pmol). Thus, we also studied the CN_*x*_ catalyst systems with the same amount of NiP and H_2_ase (200 pmol) on CN_*x*_ (5 mg) in aqueous EDTA solution (pH 4.5 and pH 7.0, respectively). At the same concentration, the enzyme (TOF=2528 h^−1^) greatly outperforms the NiP cocatalyst (TOF=64 h^−1^), demonstrating that substantial improvements are still required to develop synthetic catalysts with activities comparable to enzymes (Figure S21, Tables S1 and S3).

Finally, we photodeposited 1 wt % Pt onto CN_*x*_ (5 mg) for a direct comparison of this benchmark system with CN_*x*_–H_2_ase and CN_*x*_–NiP. Following a standard procedure,[12] the platinized CN_*x*_ system was irradiated with visible light (*λ*>420 nm) in an aqueous 10 vol % TEOA solution, generating 94 μmol H_2_ (g CN_*x*_)^−1^ h^−1^, which corresponds to a TOF_Pt_ of 4.3 mol H_2_ (mol Pt)^−1^ h^−1^. Thus, the CN_*x*_–H_2_ase and CN_*x*_–NiP systems compare favorably when using TOF as the metrics of system performance.

In summary, solar-light-driven H_2_ production with hybrid systems consisting of polymeric CN_*x*_ with H_2_ase and the bioinspired synthetic catalyst, NiP, has been demonstrated. The systems operate without a soluble redox mediator and are not limited by a photo-unstable or expensive dye. The semibiological CN_*x*_–H_2_ase assembly achieved a record TOF of 5532 h^−1^ and TON of >50 000 after two days as a cocatalyst with CN_*x*_. The additional use of the redox mediator MV allowed for the photogeneration of H_2_ with a TOF of 12.3 s^−1^ and a TON of >1×10^6^, which displays the further potential of the hybrid assembly after optimization of the biomaterial interface. CN_*x*_–H_2_ase also maintains respectable activity under visible light irradiation for more than 48 h. Recent investigations into improving the absorption profile of CN_*x*_ in the visible range demonstrate the potential of this material and illustrate that its use as a light-harvesting material will continue to develop, as its absorption profile is further improved.[21] The entirely synthetic CN_*x*_–NiP system displays an unprecedentedly high TOF (109 h^−1^) and TON (166) for a hybrid system made of a molecular cocatalyst with CN_*x*_ in purely aqueous solution. This work advances the use of hybrid photocatalytic schemes by integrating highly active electrocatalysts with the photostable and inexpensive CN_*x*_, which is shown to be compatible with biological and bioinspired electrocatalysts, namely hydrogenases and their mimics in aqueous solution.

## References

[1a] Ott S, Kritikos M, Åkermark B, Sun L Angew. Chem. Int. Ed.

[1b] Reece SY, Hamel JA, Sung K, Jarvi TD, Esswein AJ, Pijpers JJH, Nocera DG (2003). Science.

[1c] McKone JR, Lewis NS, Gray HB (2011). Chem. Mater.

[1d] Lin C-Y, Lai Y-H, Mersch D, Reisner E (2014). Chem. Sci.

[1e] Hu S, Shaner MR, Beardslee JA, Lichterman M, Brunschwig BS, Lewis NS (2012). Science.

[2a] Kudo A, Miseki Y Chem. Soc. Rev.

[2b] Chen X, Shen S, Guo L, Mao SS (2009). Chem. Rev.

[2c] Lakadamyali F, Reisner E (2010). Chem. Commun.

[2d] Tong H, Ouyang S, Bi Y, Umezawa N, Oshikiri M, Ye J (2011). Adv. Mater.

[2e] Tachibana Y, Vayssieres L, Durrant JR (2012). Nat. Photonics.

[2f] Han Z, Qiu F, Eisenberg R, Holland PL, Krauss TD (2012). Science.

[2g] Qu Y, Duan X (2012). Chem. Soc. Rev.

[2h] Wen F, Li C (2013). Acc. Chem. Res.

[2i] *Chem. Soc. Rev***2014**

[3a] Jones AK, Sillery E, Albracht SPJ, Armstrong FA Chem. Commun.

[3b] Vincent KA, Parkin A, Armstrong FA (2002). Chem. Rev.

[3c] Armstrong FA, Belsey NA, Cracknell JA, Goldet G, Parkin A, Reisner E, Vincent KA, Wait AF (2007). Chem. Soc. Rev.

[4] Sakai T, Mersch D, Reisner E Angew. Chem. Int. Ed.

[5a] Reisner E, Fontecilla-Camps JC, Armstrong FA Chem. Commun.

[5b] Reisner E, Powell DJ, Cavazza C, Fontecilla-Camps JC, Armstrong FA (2009). J. Am. Chem. Soc.

[6a] Greene BL, Joseph CA, Maroney MJ, Dyer RB J. Am. Chem. Soc.

[6b] Bachmeier A, Wang VCC, Woolerton TW, Bell S, Fontecilla-Camps JC, Can M, Ragsdale SW, Chaudhary YS, Armstrong FA (2012). J. Am. Chem. Soc.

[6c] Wilker MB, Shinopoulos KE, Brown KA, Mulder DW, King PW, Dukovic G (2013). J. Am. Chem. Soc.

[7a] Wilson AD, Newell RH, McNevin MJ, Muckerman JT, Rakowski DuBois M, DuBois DL J. Am. Chem. Soc.

[7b] Helm ML, Stewart MP, Bullock RM, Rakowski DuBois M, DuBois DL (2006). Science.

[7c] Kilgore UJ, Roberts JAS, Pool DH, Appel AM, Stewart MP, Rakowski DuBois M, Dougherty WG, Kassel WS, Bullock RM, DuBois DL (2011). J. Am. Chem. Soc.

[7d] Kilgore UJ, Stewart MP, Helm ML, Dougherty WG, Kassel WS, Rakowski DuBois M, DuBois DL, Bullock RM (2011). Inorg. Chem.

[7e] Jain A, Lense S, Linehan JC, Raugei S, Cho H, DuBois DL, Shaw WJ (2011). Inorg. Chem.

[7f] Wiese S, Kilgore UJ, DuBois DL, Bullock RM (2011). ACS Catal.

[7g] Wiese S, Kilgore UJ, Ho M-H, Raugei S, DuBois DL, Bullock RM, Helm ML (2012). ACS Catal.

[8] Berggren G, Adamska A, Lambertz C, Simmons TR, Esselborn J, Atta M, Gambarelli S, Mouesca J-M, Reijerse E, Lubitz W, Happe T, Artero V, Fontecave M (2013). Nature.

[9] Gross MA, Reynal A, Durrant JR, Reisner E (2014). J. Am. Chem. Soc.

[10a] Lotsch BV, Döblinger M, Sehnert J, Seyfarth L, Senker J, Oeckler O, Schnick W Chem. Eur. J.

[10b] Wang X, Maeda K, Thomas A, Takanabe K, Xin G, Carlsson JM, Domen K, Antonietti M (2007). Nat. Mater.

[10c] Zhang J, Chen X, Takanabe K, Maeda K, Domen K, Epping JD, Fu X, Antonietti M, Wang X (2009). Angew. Chem. Int. Ed.

[10d] Zheng Y, Liu J, Liang J, Jaroniec M, Qiao SZ (2010). Energy Environ. Sci.

[10e] Wang Y, Wang X, Antonietti M (2012). Angew. Chem. Int. Ed.

[10f] Jun Y-S, Park J, Lee SU, Thomas A, Hong WH, Stucky GD (2012). Angew. Chem. Int. Ed.

[10g] Zhang G, Zhang M, Ye X, Qiu X, Lin S, Wang X (2013). Adv. Mater.

[10h] Cao S, Yu J (2014). J. Phys. Chem. Lett.

[11a] Hong J, Wang Y, Wang Y, Zhang W, Xu R ChemSusChem.

[11b] Hou Y, Laursen AB, Zhang J, Zhang G, Zhu Y, Wang X, Dahl S, Chorkendorff I (2013). Angew. Chem. Int. Ed.

[11c] Hou Y, Zhu Y, Xu Y, Wang X (2013). Appl. Catal. B.

[12] Maeda K, Wang X, Nishihara Y, Lu D, Antonietti M, Domen K (2009). J. Phys. Chem. C.

[13] Dong J, Wang M, Li X, Chen L, He Y, Sun L (2012). ChemSusChem.

[14a] Cao S-W, Liu X-F, Yuan Y-P, Zhang Z-Y, Fang J, Loo SCJ, Barber J, Sum TC, Xue C Phys. Chem. Chem. Phys.

[14b] Li X, Ward AJ, Masters AF, Maschmeyer T (2013). Chem. Eur. J.

[14c] Song X-W, Wen H-M, Ma C-B, Cui H-H, Chen H, Chen C-N (2014). RSC Adv.

[15a] Garcin E, Vernede X, Hatchikian EC, Volbeda A, Frey M, Fontecilla-Camps JC Structure.

[15b] Parkin A, Goldet G, Cavazza C, Fontecilla-Camps JC, Armstrong FA (1999). J. Am. Chem. Soc.

[15c] Baltazar CSA, Marques MC, Soares CM, DeLacey AM, Pereira IAC, Matias PM (2008). Eur. J. Inorg. Chem.

[15d] Wombwell C, Reisner E (2014). Dalton Trans.

[16] Reisner E (2011). Eur. J. Inorg. Chem.

[17] Ishida Y, Chabanne L, Antonietti M, Shalom M (2014). Langmuir.

[18] Di Y, Wang X, Thomas A, Antonietti M (2010). ChemCatChem.

[19] Hoogvliet JC, Lievense LC, van Dijk C, Veeger C (1988). Eur. J. Biochem.

[20a] Wang D, Zhang Y, Chen W Chem. Commun.

[20b] Gimbert-Suriñach C, Albero J, Stoll T, Fortage J, Collomb M-N, Deronzier A, Palomares E, Llobet A (2014). J. Am. Chem. Soc.

[21a] Schwinghammer K, Tuffy B, Mesch MB, Wirnhier E, Martineau C, Taulelle F, Schnick W, Senker J, Lotsch BV Angew. Chem. Int. Ed.

[21b] Schwinghammer K, Mesch MB, Duppel V, Ziegler C, Senker J, Lotsch BV (2013). J. Am. Chem. Soc.

[21c] Martin DJ, Qiu K, Shevlin SA, Handoko AD, Chen X, Guo Z, Tang J (2014). Angew. Chem. Int. Ed.

[21d] Algara-Siller G, Severin N, Chong SY, Björkman T, Palgrave RG, Laybourn A, Antonietti M, Khimyak YZ, Krasheninnikov AV, Rabe JP, Kaiser U, Cooper AI, Thomas A, Bojdys MJ (2014). Angew. Chem. Int. Ed.

