# Supplementary material for: Photocatalytic Hydrogen Production using Polymeric Carbon Nitride with a Hydrogenase and a Bioinspired Synthetic Ni Catalyst
Source: Angew Chem Int Ed Engl. 2014 Sep 9;53(43):11538–42. doi: 10.1002/anie.201406811 (PMC4497605; doi:10.1002/anie.201406811)
Supplement: Supplementary file 1 [file anie0053-11538-sd1.pdf]

Angewandte Chemie
Eine Zeitschrift der Gesellschaft Deutscher Chemiker

Supporting Information
© Wiley-VCH 2014
69451 Weinheim, Germany

Photocatalytic Hydrogen Production using Polymeric Carbon Nitride with a Hydrogenase and a Bioinspired Synthetic Ni Catalyst**

*Christine A. Caputo, Manuela A. Gross, Vincent W. Lau, Christine Cavazza, Bettina V. Lotsch, and Erwin Reisner**

anie_201406811_sm_miscellaneous_information.pdf

Supporting Information

Table of Contents:

	PAGE
Experimental Section	S2
References	S5
Tables S1 to S4	S6
Figures S1 to S21	S10

Experimental Section

Materials. Chemicals were purchased from commercial suppliers and used without further purification. The [NiFeSe]-hydrogenase from *Desulfomicrobium baculatum* (*Dmb* [NiFeSe]-H_2ase) was purified under air by a previously published method.[1] The pure enzyme was dialyzed against 20 mM Tris/HCl, pH 7.6. The enzyme integrity was verified by measuring its specific activity with an aliquot of the H_2ase under H_2 in the presence of 1 mM methylviologen (MV) for 30 min at 30 °C. H_2 oxidation activity was determined spectrophotometrically at 604 nm by following the color change of oxidized MV in a hydrogen-saturated solution, after adding enzyme. The preparation has a specific activity of 2115 µmol H_2 min^{-1} mg^{-1},[2] and the stock enzyme solution was diluted with an aqueous TEOA solution (0.1 M, pH 7) before photocatalytic experiments in an anaerobic glovebox. CN_x (melon)[3] and NiP[4] were synthesized as previously described. Reagents for the analytical part of the work were of the highest available purity.

Photocatalysis Experiments. A standard photocatalytic experimental set-up was used as follows: Melon (CN_x) was added to a borosilicate glass tube containing a magnetic stir bar (total volume 7.74 mL). An aqueous solution of electron donor (usually 0.1 M) was then added along with the catalyst being used (NiP), sealed with a small septum. The suspension was then sonicated for 20 min under air. The suspension was then purged for 20 min with 2% CH_4 in N_2. In the case of hydrogenase experiments, the enzyme was added after purging and then the vial was purged with 2% CH_4 in N_2 for an additional 5 min. The vials were then placed in a water-jacketed rack and irradiated with 1 Sun using a W-halogen lamp (Newport Oriel Solar Light Simulator, 1000 W, 100 mW cm^{-2}) with an air mass 1.5 global (AM 1.5G) filter in the absence or presence of a 420 nm UV-broad band filter (UQG Optics), with stirring. Headspace gases were sampled using Hamilton air-tight syringes by injecting 20 µL into the gas chromatograph (Agilent 7890A Series GC equipped with a 5 Å molecular sieve column; oven held at 45 °C) at regular intervals. H_2 produced was quantified by comparison to the CH_4 internal standard and each measurement was carried out in triplicate.

Synthesis and activity of platinized CN_x (CN_x-Pt).

We prepared a standard CN_x-Pt suspension (1 % wt. Pt) to benchmark CN_x-H_2ase and CN_x-NiP. Following a published procedure,[5] we added 1 wt.% Pt (4.6 µL of a 1 wt% H_2PtCl_6 solution in 0.1 M TEOA at pH 7) to CN_x (5 mg) in 10 vol. % TEOA (3 mL, pH 10.6). The vial was sonicated for 20 min and the headspace purged with 2 % CH_4/N_2. The photoreactor was then irradiated (1 sun, 100 mW, λ > 420 nm) and the platinum

photodeposited *in situ*. After 1 h, 94 µmol H_2 (g CN_x)$^{-1}$ h^{-1} and a TOF_{Pt} 4.3 h^{-1} were obtained.

Centrifugation Experiments. Photocatalytic experiments were set up under standard conditions and were irradiated with 1 Sun for 2 h and H_2 production monitored by GC. The suspension was then transferred (in air) to centrifuge tubes and spun down for 5 min (5000 rpm). The supernatant was decanted and the pellet washed with distilled water (3 mL). The sample was centrifuged again for 5 min (5000 rpm) and supernatant decanted. The melon pellet was resuspended in 0.1 M EDTA (NiP, pH 4.5; H_2ase pH 6) and vortexed for 2 min. The suspension was returned to the borosilicate glass vials, sealed and purged with 2% CH_4 in N_2 for 10 min. The vials were irradiated under 1 Sun for and additional 2 h and H_2 production monitored by GC.

UV-Vis Spectrophotometry. UV-Vis spectra of the NiP solution (6.7 µM) in 0.1 M EDTA at pH 4.5 was measured with a Varian Cary 50 UV-vis spectrophotometer using quartz cuvettes. Polymeric carbon nitride was added to this solution (3 mL, 5 mg) and sonicated for 20 min. The suspension was then centrifuged (Eppendorf Centrifuge 5804) and a UV-Vis spectrum of the supernatant was recorded. The difference in absorbance before and after treatment with melon was calculated at λ = 329 and 450 nm and the resulting difference % averaged. A loading of 0.76 nmol NiP (mg C_3N_4)$^{-1}$ was calculated using this method.

Photoluminescence Experiments.

A vial containing 2 mg of CN_x in 0.1 M EDTA (pH 6) was sonicated for 20 min. The photoluminescence emission spectrum of the suspension was measured on a Agilent Technologies Cary Fluorescence Spectrophotometer with excitation at 365 nm. The cuvette was sonicated for 1 min to prevent aggregation and settling of the CN_x between measurements, then either MV (26 and 50 pmol) or H_2ase (33 and 50 pmol) was added and the cuvette shaken prior to measurements.

Treatment of Data. All analytical measurements were performed in triplicate. The data were treated as follows: for a sample of *n* observations x_i, the unweighted mean value x_0 and the standard deviation σ were calculated using the equations

$$x_0 = \sum_i \frac{x_i}{n} \qquad \sigma = \sqrt{\sum_i \frac{(x_i - x_0)}{(n-1)}}$$

A minimum σ of 10% was assumed for all experiments even where triplicate runs gave a weighted mean of less than 10%. The solar light source and the gas chromatograph were calibrated regularly to ensure reproducibility.

Calculation of External Quantum Efficiency (EQE). Hydrogen generation was driven by blue light (λ = 460 nm) of intensity I = 3.5 mW cm^{-2} and UV light (λ = 365 nm) of intensity I = 47 mW cm^{-2} at 25 °C from an LED light source controlled by a CompactStat, IVIUM potentiostat. The EQE can be calculated with the following formula:

$$\mathrm{EQE}\ (\%) = (2 \cdot n_{H2} \cdot N_A \cdot h \cdot c)/(t_{irr} \cdot \lambda \cdot I \cdot A) \cdot 100$$

Where n_{H2} is the moles of H_2 photo-generated, N_A is the Avogardo constant, h is the Planck constant, c is the speed of light, t_{irr} is the irradiation time, A is the irradiated area of the photoreactor.

Characterization Details of CN_x. Diffuse reflectance UV-Vis spectra were collected on a Cary 5000 spectrometer (referenced to PTFE or barium sulfate) and the spectra in percentage reflectance were converted using the Kubelka Munk function. X-ray diffraction patterns were collected using a STOE Stadi P diffractometer (Cu $K_{\alpha 1}$) in transmission mode. ATR-IR spectra were collected with a PerkinElmer UATR TWO spectrometer equipped with a diamond crystal. Surface areas were calculated using Brunauer–Emmett–Teller (BET) theory from the adsorption isotherms of the samples. Samples were heated for 6–12 h overnight at 100 °C to a vacuum of 10^{-7} mbar. Isotherms were collected on a Quantachrome Autosorb iQ gas sorption analyzer using argon as the sorbent at 87.45 K. Multipoint BET calculations were carried with the BET Assistant in the ASiQwin software, using data points from the argon adsorption isotherm at or below the maximum in $V \cdot (1\ P/P_0)$ in accordance with the ISO recommendations. All characterization data was compared to previously reported data.[3] Scanning electron microscopy (SEM) was performed on a Vega TS 5130MM (Tescan) microscope. Sample was deposited onto a carbon tab (Leco) and sputtered with gold for imaging. Zeta potential was measured using a Malvern Zetasizer Nano ZS. Sample was dispersed with sonication in NaCl solution (10 mM) of different pH (adjusted with HCl or NaOH) and allowed to stand prior to measurements in disposable cuvettes (Malvern). Measurements were conducted as four replicates; average results were quoted using the standard deviation as the error.

References

[1] A. Volbeda, P. Amara, M. Iannello, A. L. De Lacey, C. Cavazza, J. C. Fontecilla-Camps, *Chem. Commun.* **2013**, *49*, 7061-7063.

[2] E. C. Hatchikian, M. Bruschi, J. Le Gall, *Biochem. Biophys. Res. Commun.* **1978**, *82*, 451-461.

[3] X. Wang, K. Maeda, A. Thomas, K. Takanabe, G. Xin, J. M. Carlsson, K. Domen, M. Antonietti, *Nat. Mater.* **2009**, *8*, 76-80.

[4] M. A. Gross, A. Reynal, J. R. Durrant, E. Reisner, *J. Am. Chem. Soc.* **2014**, *136*, 356-366.

[5] K. Maeda, X. Wang, Y. Nishihara, D. Lu, M. Antonietti, K. Domen, *J. Phys. Chem. C.* **2009**, *113*, 4940-4947.

[6] Y. Di, X. Wang, A. Thomas, M. Antonietti, *ChemCatChem* **2010**, *2*, 834-838.

Table S1. Solar light driven H_2 production with *Dmb* [NiFeSe]-H_2ase and CN_x. Experiments were performed using CN_x (5 mg) in aqueous ascorbic acid (AA; 0.1 M), EDTA (0.1 M), TEOA (0.1 M) or potassium phosphate (KPi) solution with H_2ase as a catalyst. All experiments were carried out under standard conditions (visible light irradiation 100 mW cm^{-2}, $\lambda > 300$ nm, 25 °C, under an 2 % CH_4 in N_2 atmosphere and a solvent volume of 3 mL, with a headspace volume of 4.74 mL). Entry 5 displays 'standard conditions'.

Entry	H_2ase / pmol	pH of electron donor solution	Electron donor solution/ 0.1 M	TON (4h) ± σ / mol H_2 H_2ase^{-1}	H_2 ± σ / µmol (after 4 h)	Activity / µmol H_2 (g CN_x)$^{-1}$ h^{-1} (after 1 h)	TOF ± σ / h^{-1} (after 1 h)
**Electron Donor Dependence***							
**1***	50	6	AA	664 ± 66	0.03 ± 0.01	5.1 ± 0.5	512 ±51
**2***	50	7	TEOA	2678 ± 268	0.13 ± 0.01	11.4 ± 1.1	1135 ±117
**3***	50	7	EDTA	6482 ± 648	0.32 ±0.03	22.5 ± 3.5	2248 ± 225
**pH Dependence**							
**4**	50	5	EDTA	11637 ± 1164	0.58 ± 0.05	46.4 ± 4.6	4643 ± 464
**5**	50	6	EDTA	16466 ± 1646	0.82 ± 0.08	55.3 ± 5.5	5532 ± 553
**6**	50	7	EDTA	9135 ± 914	0.45 ± 0.05	32.1 ± 3.5	3208 ± 362
**7**	50	8	EDTA	2159 ± 216	0.11 ± 0.01	9.97 ± 3.19	997 ± 100
***Dmb* [NiFeSe] H_2ase Dependence**							
**8**	50	7	EDTA	9135 ± 914	0.45 ± 0.04	32.1 ± 3.5	3208 ±362
**9**	100	7	EDTA	8505 ± 1883	0.85 ± 0.09	55.2 ± 7.8	2761 ± 776
**10**	200	7	EDTA	8878 ± 1837	1.78 ± 0.18	101.1 ± 10.1	2528 ± 441
**Visible Light Only Irradiation (λ > 420 nm)**							
**11**	50	6	EDTA	2376 ± 267	0.12 ± 0.01	7.68 ± 0.54	768 ± 77
**Control experiments**							
**12**	0	6	EDTA	—#	—	—	—
**13***	50	6	KPi†	1096 ± 138	0.06 ±0.01	6.58 ± 0.07	658 ± 66
**14***	50; no CN_x	7	EDTA	—	—	—	—

*These experiments were carried out with a light source with lower intensity light irradiation and were only used for initial screening experiments
#No H_2 detected by GC measurements (limit of detection < 0.01%).
†A small amount of sacrificial electron donor TEOA was present in this experiment (16.5 μL of 0.1 M TEOA H_2ase buffer solution).

Table S2. Photocatalytic H_2 production using CN_x (5 mg) in aqueous EDTA (0.1 M, pH 6) solution with *Dmb* [NiFeSe]-H_2ase (50 pmol) under standard conditions with the addition of neutral density filters (50% absorbance, 80 % absorbance and no additional filter).

Entry	TON (1h) ± σ / mol H_2 H_2ase^{-1}	H_2 produced ± σ / μmol (after 1 h)	Activity / μmol H_2 (g CN_x)$^{-1}$ h^{-1} (after 1 h)	TOF ± σ / h^{-1} (after 1 h)
**No additional neutral density filter was employed**				
**1**	3323 ± 360	0.16 ± 0.02	33.2 ± 3.6	3323 ±360
**A neutral density filter (Absorbance 50%) was employed**				
**2**	3081 ± 362	0.15 ± 0.02	30.8 ± 3.6	3081 ± 362
**A neutral density filter (Absorbance 80%) was employed**				
**3**	1840 ± 184	0.09 ± 0.01	18.4 ± 1.8	1840 ± 184

*Vials were placed 10 cm closer to the light source thus may have been irradiated with a higher intensity of light than that used in standard experiments, thus a control with no filter was also measured. These measurements were performed in duplicate.

Table S3. Solar light driven H_2 production using NiP with CN_x (5 mg) in aqueous AA (0.1 M), EDTA (0.1 M), TEOA (0.1 M) or potassium phosphate (KPi) solution. All experiments were carried out under standard conditions (visible light irradiation 100 mW cm^{-2}, λ > 300 nm, 25 °C, under a 2% CH_4 in N_2 atmosphere and a solvent volume of 3 mL, with a headspace volume of 4.74 mL).

Entry	NiP / nmol	pH of electron donor solution	Electron donor solution / 0.1 M	TON ± σ / mol H_2 NiP^{-1} (after 4 h)	H_2 produced ± σ / μmol (after 4 h)	Activity / μmol H_2 (g CN_x)$^{-1}$ h^{-1} (after 1 h)	TOF ± σ / h^{-1} (after 1 h)
**ED Dependence***							
1*	50	4.5	AA	0.8 ± 0.1	0.04 ± 0.01	—	—
2*	50	4.5	EDTA	14.2 ± 0.18	0.71 ± 0.07	34.2 ± 2.2	3.4 ± 0.03
3*	50	7	EDTA	0.8 ± 0.2	0.04 ± 0.01	7.6 ± 0.8	0.76 ± 0.07
4*	50	N/A	MeOH (with 1% H_2O)	2.34 ± 0.55	0.12 ± 0.03	6.7 ± 1.1	0.67 ±0.07
5*	50	4.5	TEOA	0.58 ± 0.05	0.03 ± 0.01	5.9 ± 1.2	0.59 ± 0.05
6*	50	7	TEOA	2.4 ± 0.4	0.11 ± 0.02	7.5 ± 0.7	0.75 ±0.07
**pH Dependence**							
7	20	3.5	EDTA	138.5 ± 13.8	2.77 ± 0.27	422.3 ± 42.2	105.6 ± 10.6
8	20	4.5	EDTA	166.1 ± 20.6	3.32 ± 0.41	437.1 ± 43.7	109.3 ± 10.9
9	20	5.5	EDTA	129.9 ± 20.1	2.59 ± 0.43	297.7 ± 34.8	74.4 ± 8.7
**NiP Dependence**							
10	0.2	4.5	EDTA	289.6 ± 29	0.057 ± 0.003	2.56 ± 0.25	64.1 ± 6.4
11	10	4.5	EDTA	17.2 ± 0.9	1.72 ± 0.17	239.2 ± 16.4	12.0 ± 1.2
12	20	4.5	EDTA	166.1 ± 20.6	3.32 ± 0.41	437.1 ± 43.7	109.3 ± 10.9
13	50	4.5	EDTA	107.2 ± 10.7	5.36 ± 0.54	445.8 ± 44.6	45.0 ± 4.5
14	100	4.5	EDTA	69.9 ± 7.0	6.99 ± 0.70	526.0 ± 24.4	26.3 ± 2.6
15	300	4.5	EDTA	86.6 ± 8.6	8.66 ± 0.86	416.2 ± 21.8	22.8 ± 2.3
**Visible Light Only Irradiation (λ > 420 nm)**							
16	50	4.5	EDTA	60.0 ± 6.0	1.20 ± 0.12	73.4± 7.3	18.3 ± 1.8
**Control Experiments**							
17	0	4.5	EDTA	—	0.10 ± 0.01	—	—
18*	50	4.5	KPi buffer	4.9 ± 1.7	0.10 ± 0.04	18.6 ± 1.62	4.7 ± 0.4
19*	50 –no CN_x	4.5	EDTA	—	—#	—	—

*These experiments were carried out with a non-calibrated solar light simulator and the intensity is therefore not precisely AM 1.5G as for the other experiments (note that entries 1-6 were irradiated with similar light intensity and the values for 1-6 in the Table are therefore comparable). # No H_2 detected by GC measurements (limit of detection <0.01 %)

Table S4. Photocatalytic H_2 production using CN_x (5 mg) in aqueous EDTA (0.1 M, pH 4.5) solution with NiP (20 nmol) under standard conditions with the addition of neutral density filters (50% absorbance, 80% absorbance and no filter).

Entry	TON (1h) ± σ / mol H_2 mol NiP^{-1}	H_2 produced ± σ / µmol (after 1 h)	Activity / µmol H_2 (g CN_x)$^{-1}$ h^{-1} (after 1 h)	TOF ± σ / h^{-1} (after 1 h)
**No additional neutral density filter was employed**				
1*	71.1 ± 5.3	1.42 ± 0.14	284.5 ± 28.5	71.1 ± 7.1
**A neutral density filter (Absorbance 50%) was employed**				
2*	32.4 ± 3.1	0.65 ± 0.01	129.6 ± 1.3	32.4± 3.2
**A neutral density filter (Absorbance 80%) was employed**				
3*	13.1 ± 1.4	0.26 ± 0.03	52.7 ± 5.3	13.1 ±1.4

*These experiments were carried out with a non-calibrated solar light simulator and the intensity is therefore not precisely AM 1.5G as for the other experiments (note that entries 1-3 were irradiated with similar light intensity and the values in the Table are therefore comparable). These measurements were performed in duplicate.

.

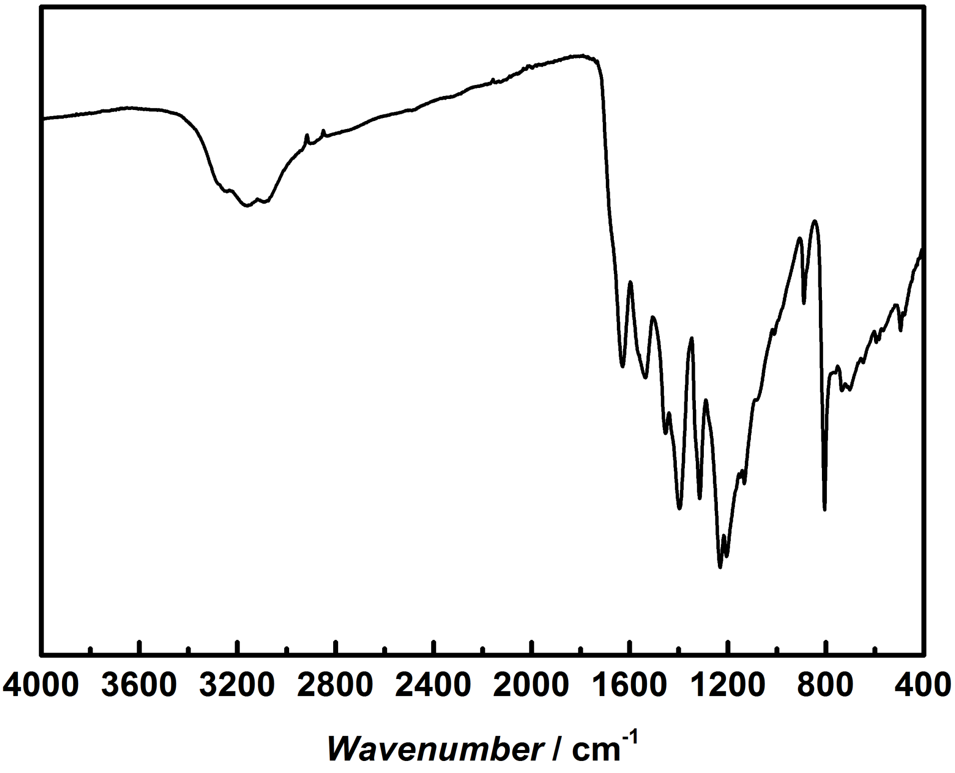

Figure S1. FT-IR spectrum of CN_x.

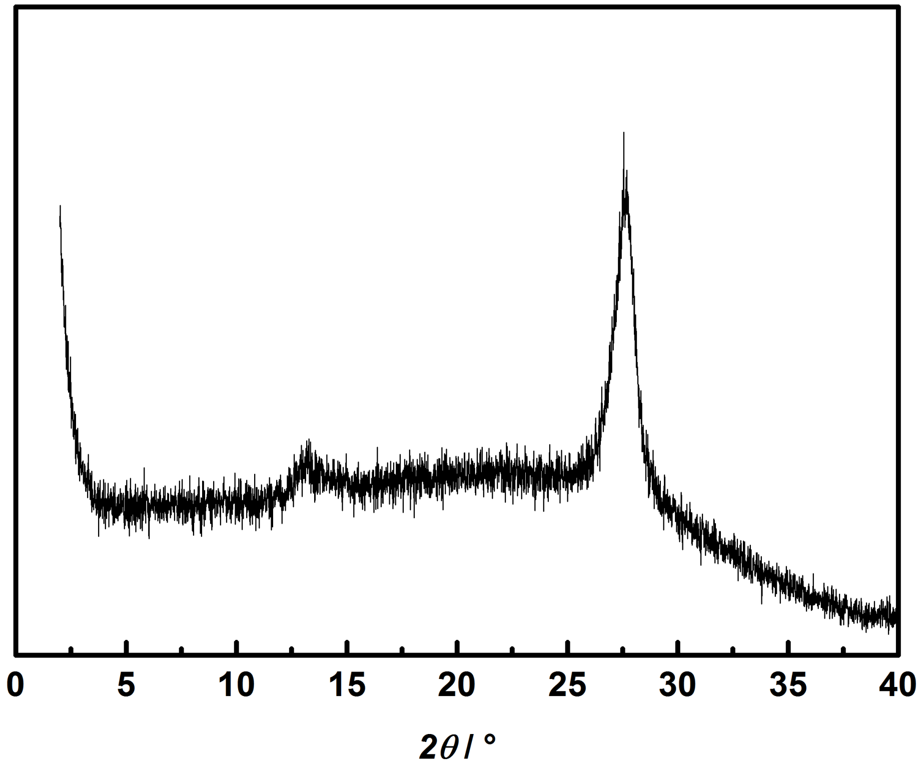

Figure S2. XRD pattern of CN_x.

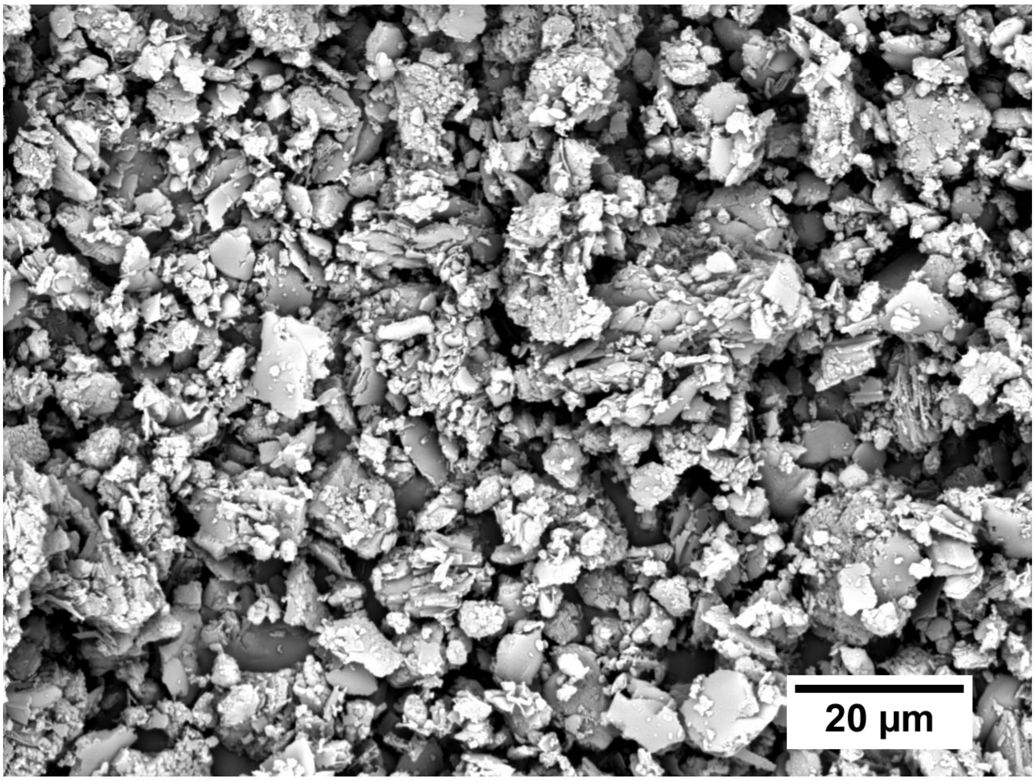

Figure S3. SEM image of CN_x.

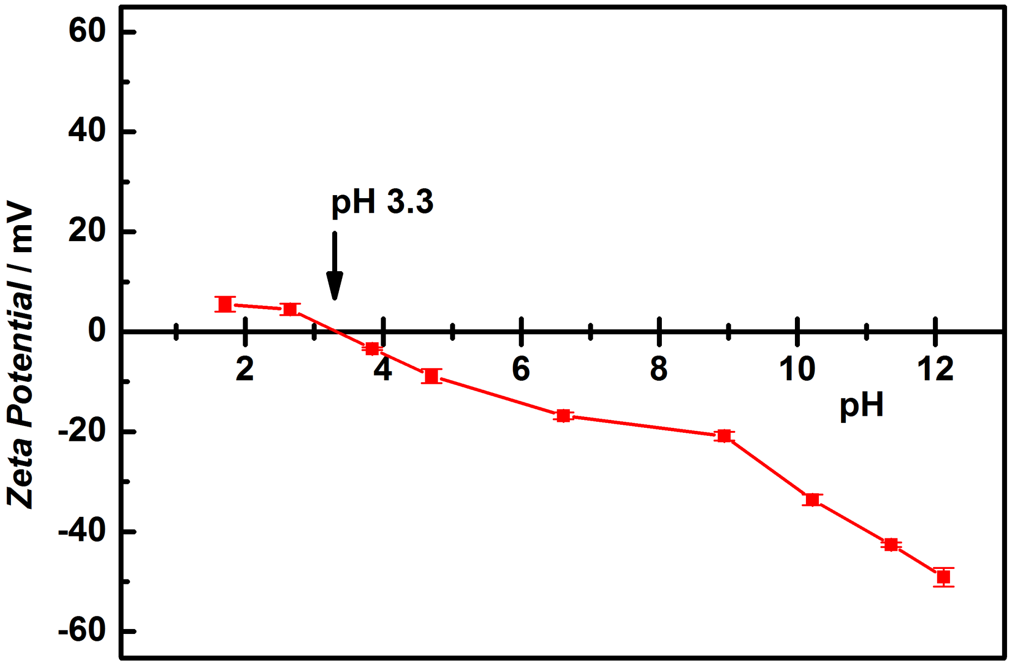

Figure S4. Determination of the isoelectric point of CN_x using a zeta potential *vs* pH plot. The isoelectric point was found to be at around 3.3, which is in broad agreement with the value of 4.1 measured in a previous publication.[6]

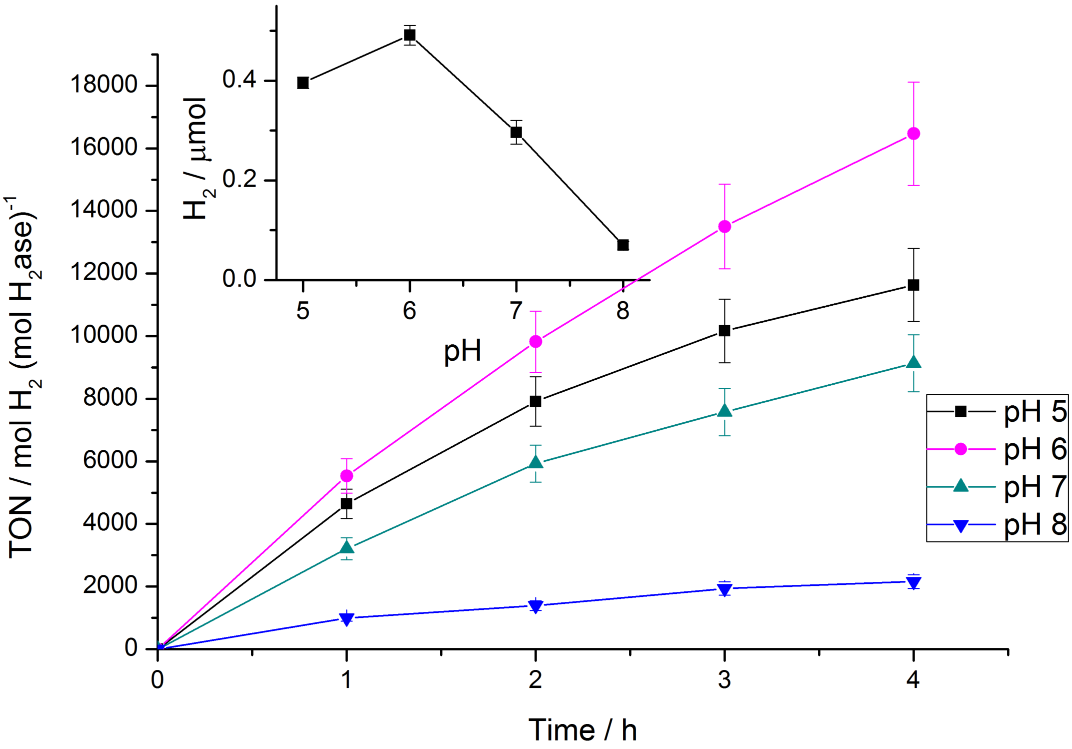

Figure S5. TON_{H2ase} for photo-H_2 production with *Dmb* [NiFeSe]-H_2ase (50 pmol) in an aqueous EDTA solution (0.1 M) with CN_x (5 mg) at various pH values (1 sun irradiation; 100 mW cm^{-2}, λ > 300 nm, 25 °C). Inset: H_2 production with varying pH after 2 h of irradiation.

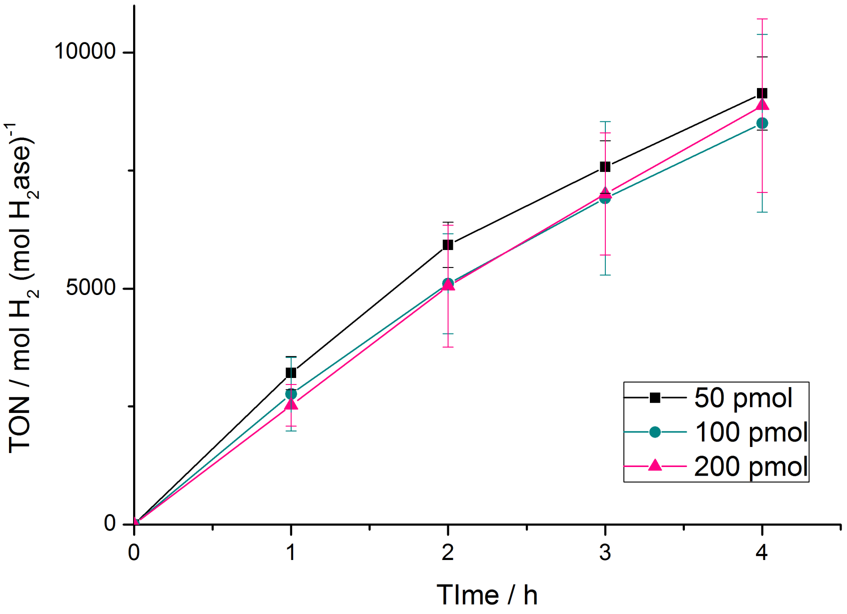

Figure S6. Photocatalytic TON_{H2ase} in aqueous EDTA solution (0.1 M, pH 7) with different amounts of *Dmb* [NiFeSe]-H_2ase and 5 mg CN_x (1 sun irradiation; 100 mW cm^{-2}, λ > 300 nm, 25 °C).

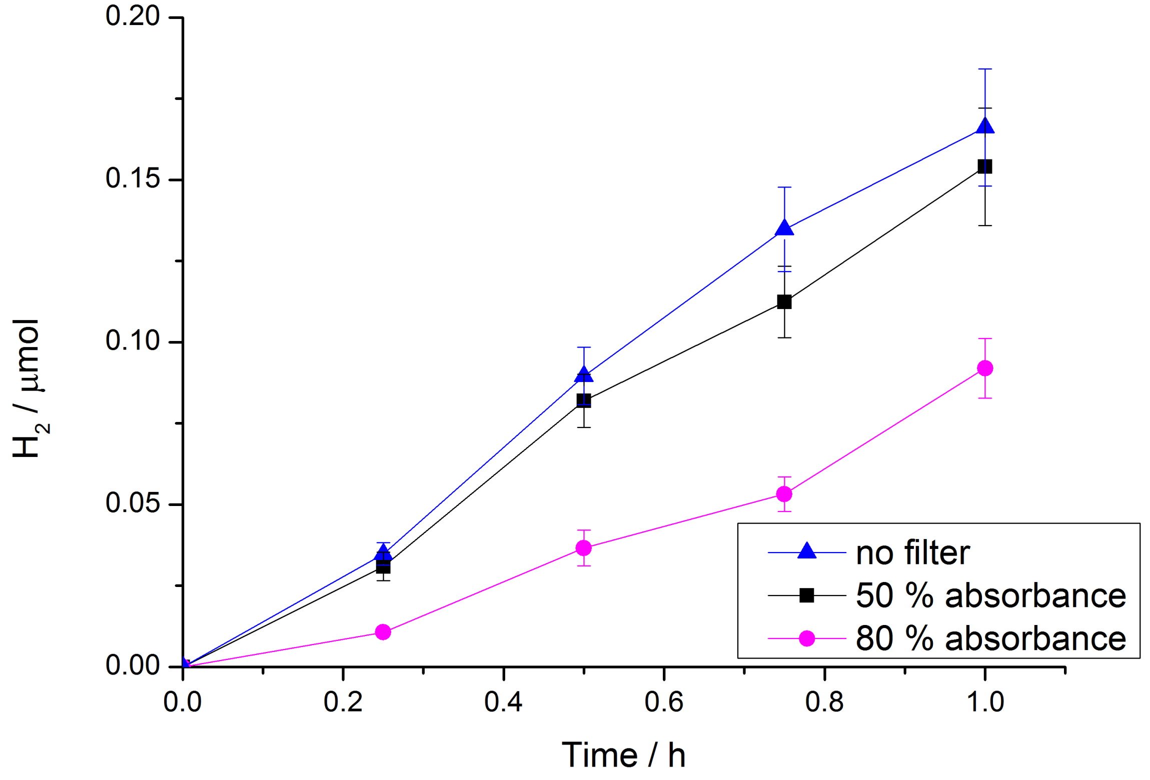

Figure S7. Photo-H_2 production using CN_x (5 mg) in aqueous EDTA solution (0.1 M, pH 6) with *Dmb* [NiFeSe]-H_2ase (50 pmol) under 1 sun irradiation (100 mW cm^{-2}, $\lambda > 300$ nm, 25 °C) with the addition of neutral density filters absorbing 50% and 80% of the incident light.

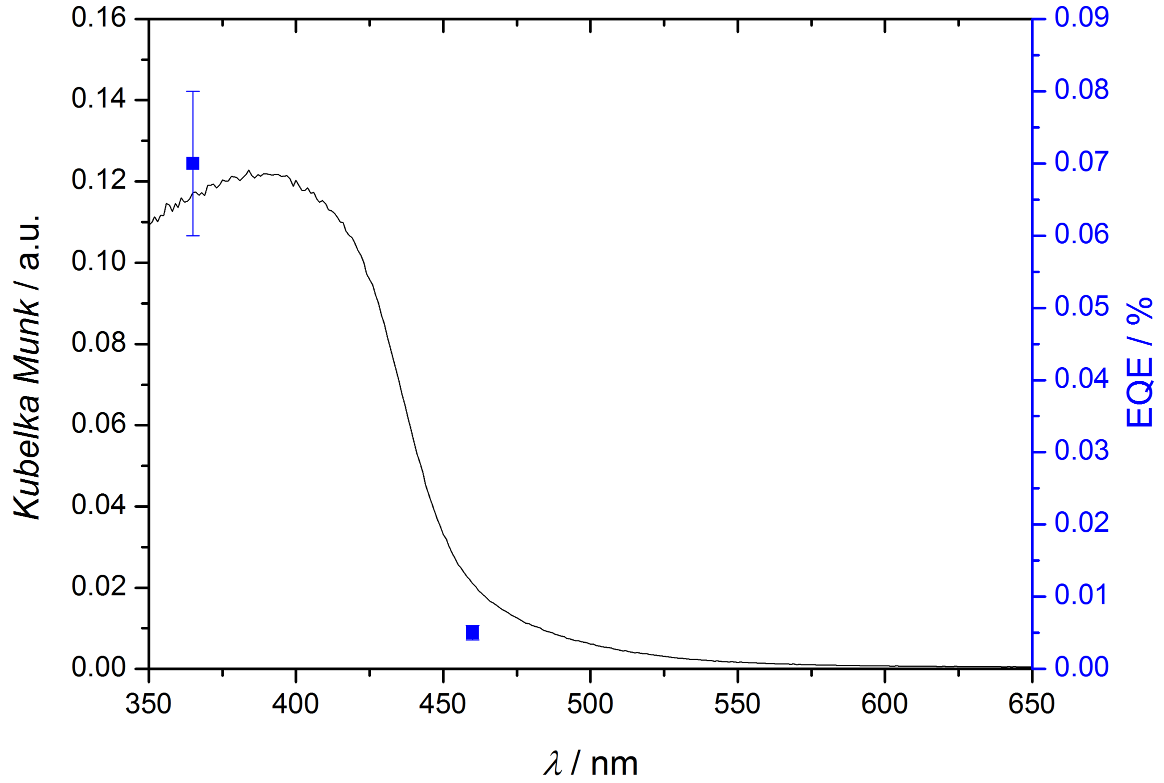

Figure S8. Activity plot for CN_x-H_2ase. Diffuse reflectance UV-vis spectrum with EQE values determined at λ = 365 and 460 nm for H_2ase. Conditions used to determine EQE: CN_x (5 mg) in aqueous EDTA solution (0.1 M, pH 6) with *Dmb* [NiFeSe]-H_2ase (50 pmol) at 25 °C.

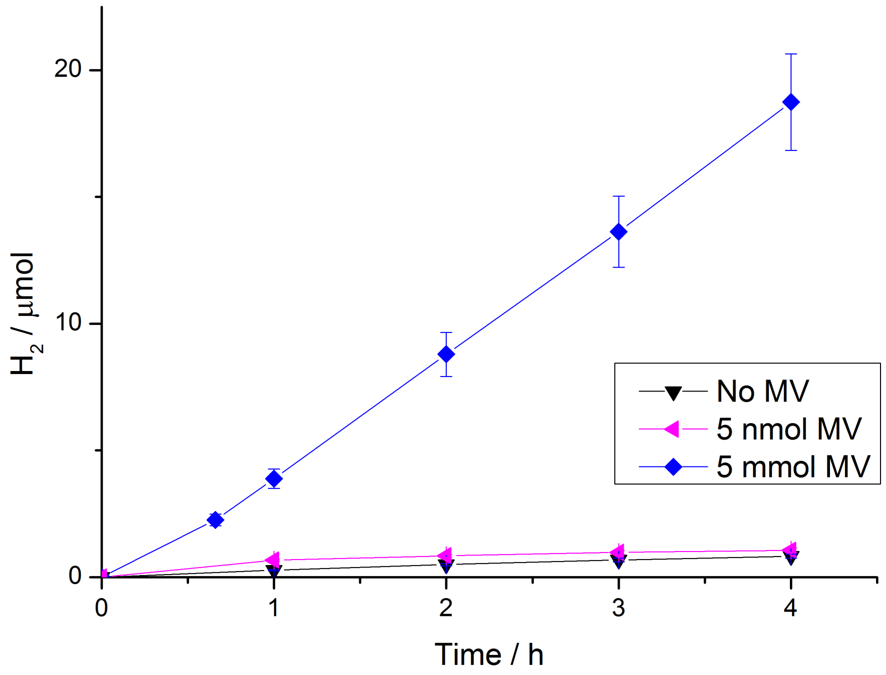

Figure S9. Photo-H_2 production with *Dmb* [NiFeSe]-H_2ase (50 pmol) with CN_x (5 mg) in aqueous EDTA solution (0.1 M, pH 6) in the presence of 5 nmol (1000 equivalents) and 5 mmol (100,000 equivalents) of methyl viologen (MV) (1 sun irradiation; 100 mW cm^{-2}, λ > 300 nm, 25 °C).

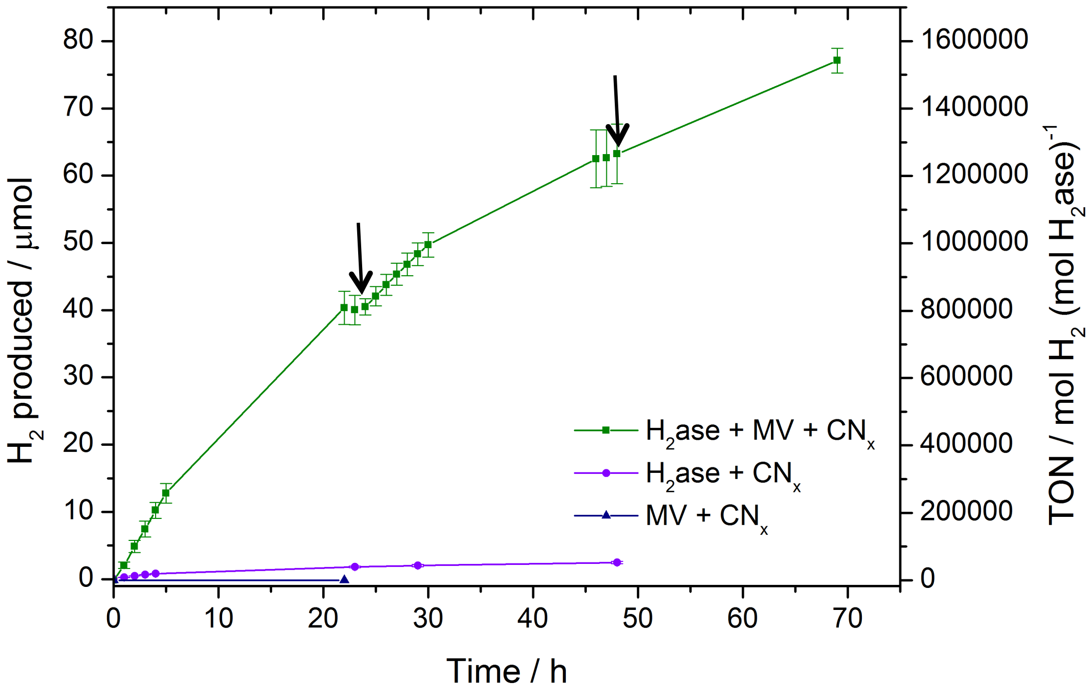

Figure S10. Long-term photo-H_2 production with *Dmb* [NiFeSe]-H_2ase (50 pmol) and CN_x (5 mg) in aqueous EDTA solution (0.1 M, pH 6) in the presence and absence of methyl viologen (MV; 5 µmol) under 1 sun irradiation (100 mW cm^{-2}, λ > 300 nm, 25 °C). The photoreactor was purged with 2 % CH_4/N_2 after 24 and 48 h and additional MV (5 µmol) was added (indicated by arrows). A control experiment with MV and CN_x but no H_2ase showed negligible H_2 after 24 h.

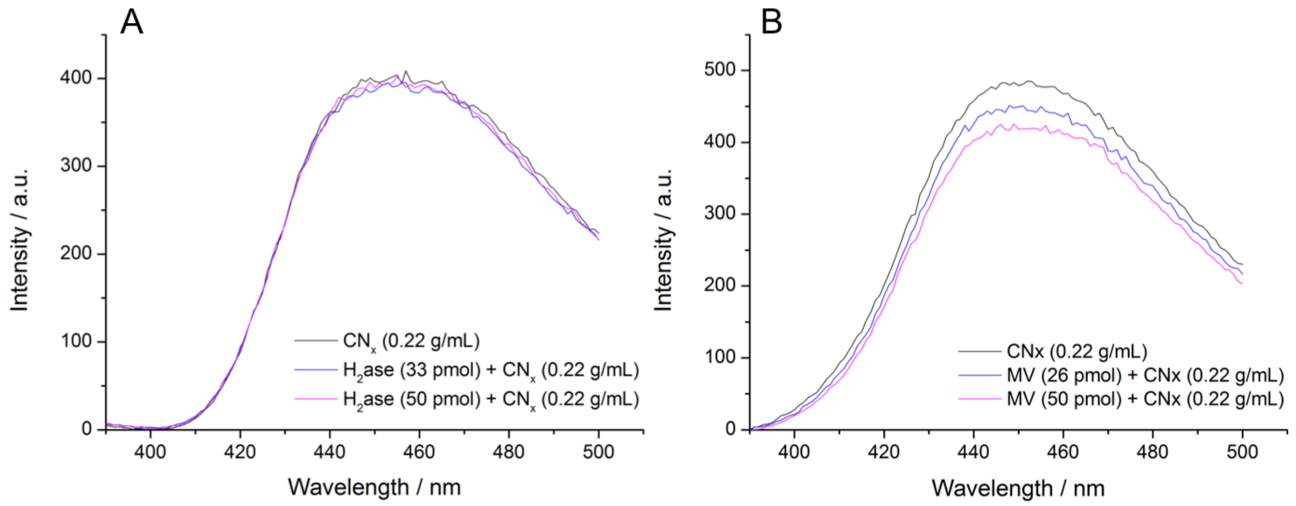

Figure S11. Steady-state photoluminescence spectra (excitation λ = 365 nm). (A) Addition of H_2ase (33 and 50 pmol) to a suspension of CN_x (0.22 g mL^{-1} 0.1 M EDTA pH 6). (B) Addition of MV (26 pmol, 50 pmol) to a suspension of CN_x (0.22 g mL^{-1} 0.1 M EDTA pH 6).

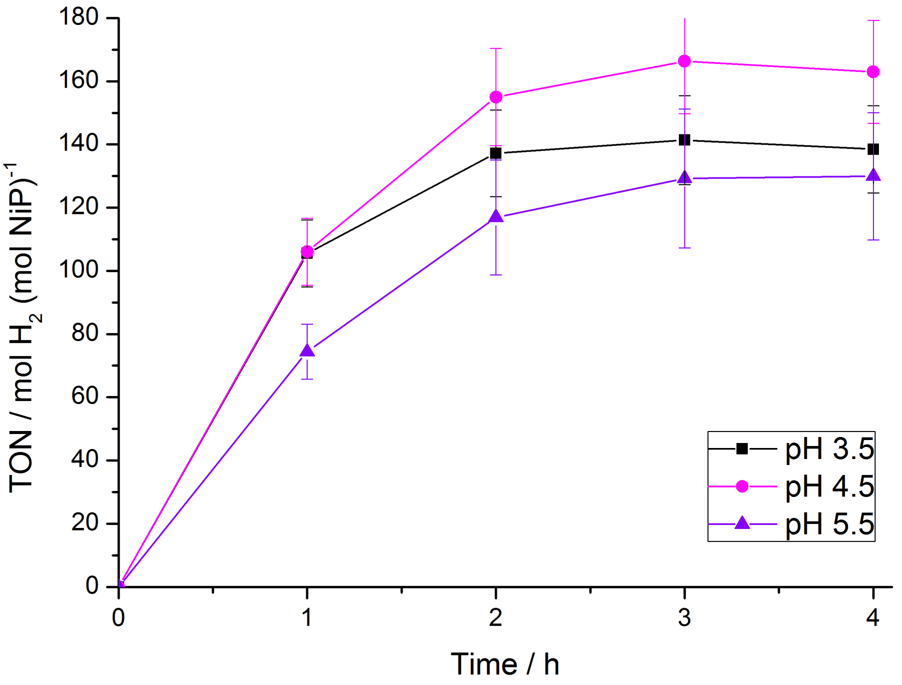

Figure S12. TON_{NiP} vs. pH for photo-H_2 production with NiP (20 nmol) with CN_x (5 mg) in aqueous EDTA aqueous solution (0.1 M, pH 4.5) under 1 sun irradiation (100 mW cm^{-2}, λ > 300 nm, 25 °C).

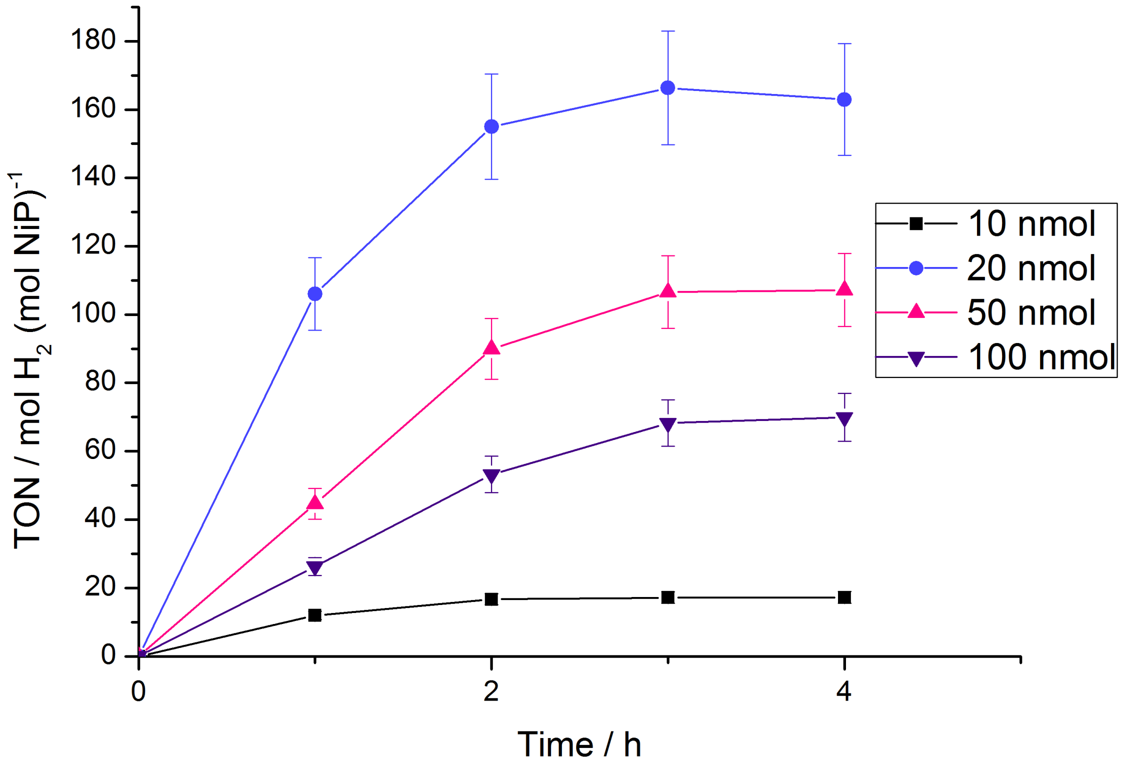

Figure S13. TON_{NiP} from solar irradiation of CN_x-NiP with varying amounts of NiP with CN_x (5 mg) in aqueous EDTA solution (0.1 M, pH 4.5) under 1 sun irradiation(100 mW cm^{-2}, $\lambda > 300$ nm, 25 °C).

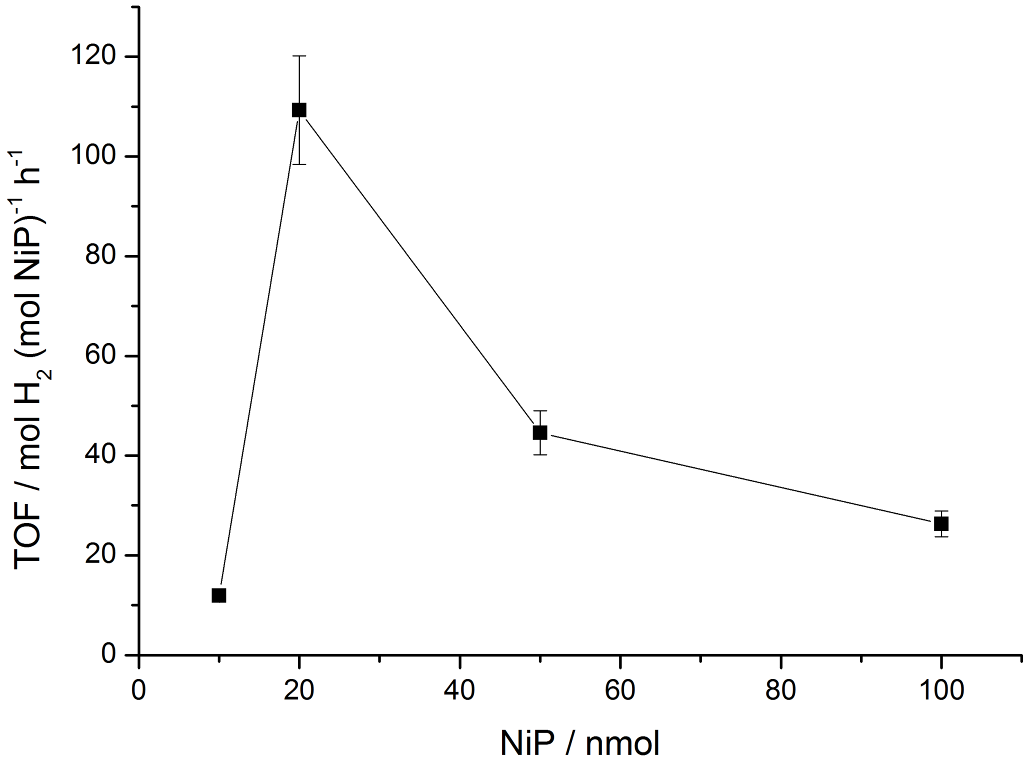

Figure S14. TOF_{NiP} in irradiated NiP-CN_x with varying amounts of NiP with CN_x (5 mg) in aqueous EDTA solution (0.1 M, pH 4.5) under 1 sun irradiation (100 mW cm^{-2}, $\lambda > 300$ nm, 25 °C).

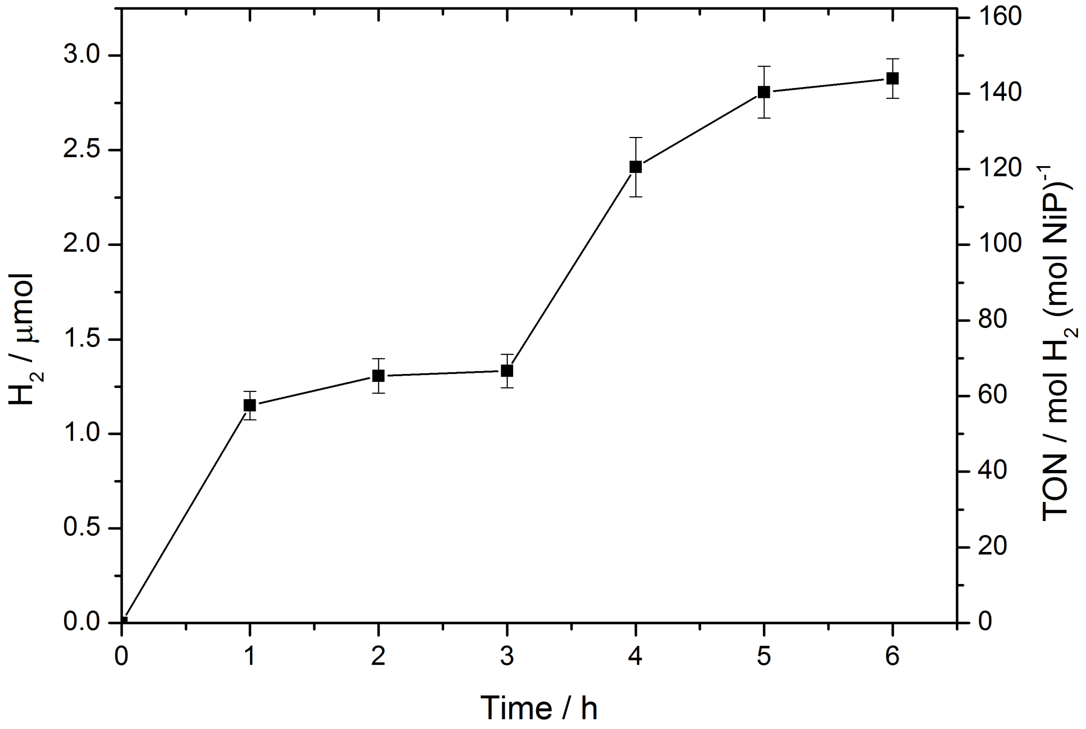

Figure S15. Photo-H_2 production with NiP (20 nmol) with CN_x (5 mg) in aqueous EDTA solution (0.1 M, pH 4.5) under 1 sun irradiation (100 mW cm^{-2}, λ > 300 nm, 25 °C). Additional NiP (20 nmol) was added after 3 h.

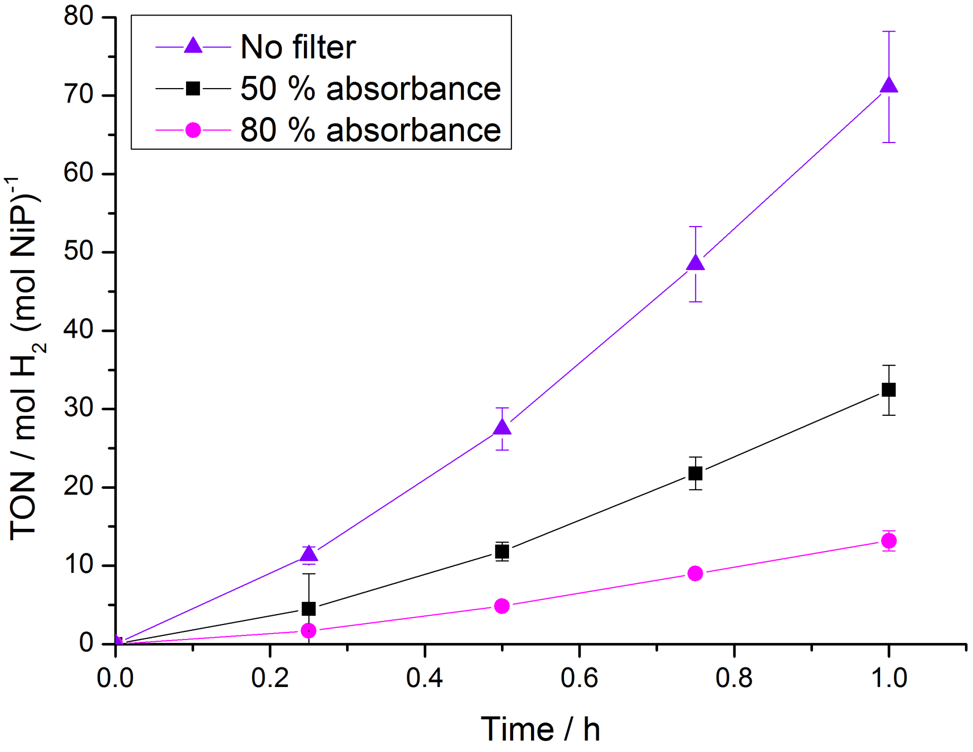

Figure S16. Hydrogen Production using NiP (20 nmol) with CN_x (5 mg) in aqueous EDTA solution (0.1 M, pH 4.5) under 1 sun irradiation (100 mW cm^{-2}, λ > 300 nm, 25 °C) with the addition of neutral density filters absorbing 50 % and 80 % of the incident light.

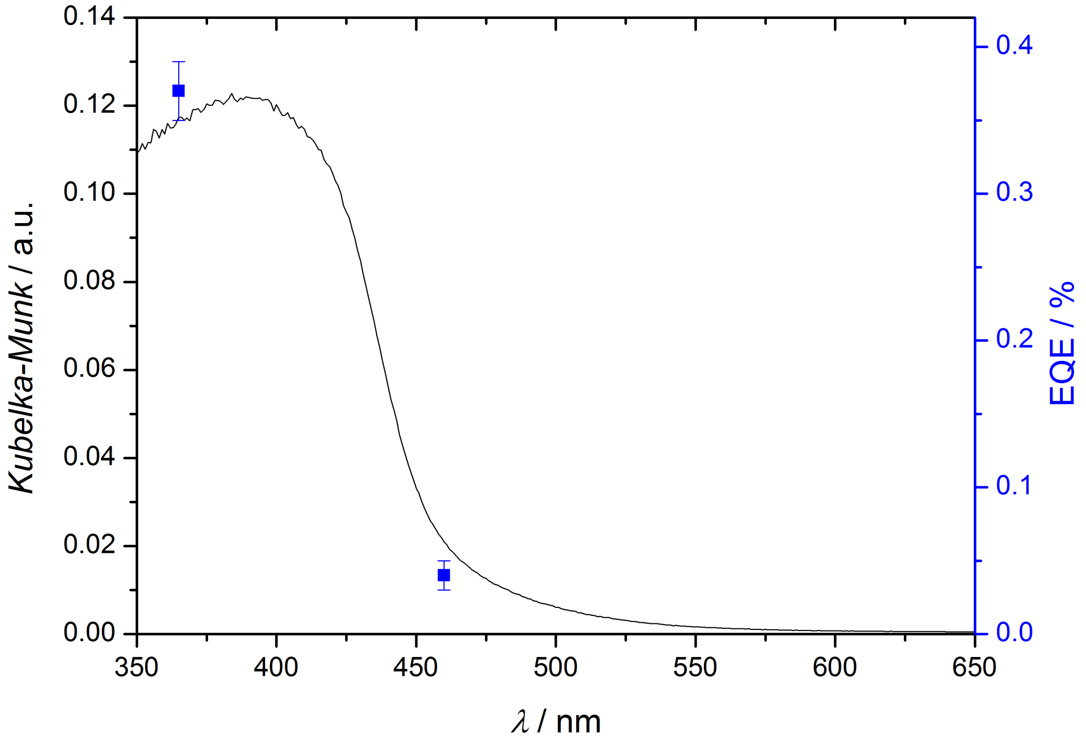

Figure S17. Activity plot for NiP-CN_x. Diffuse reflectance UV-vis spectrum overlaid with EQE values (%) determined at λ = 365 and 460 nm for NiP. Conditions used: CN_x (5 mg) in aqueous EDTA solution (0.1 M, pH 6) with NiP (20 nmol) at 25 °C.

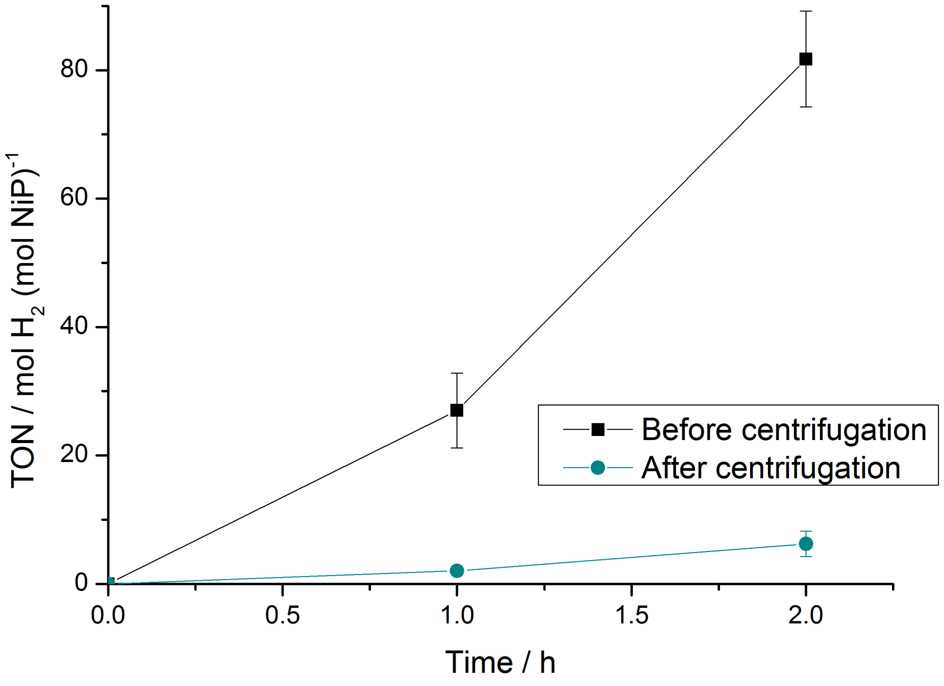

Figure S18. Centrifugation test using NiP (20 nmol) with CN_x (5 mg) in aqueous EDTA solution (0.1 M, pH 4.5) (< 1 sun irradiation with a non-calibrated solar simulator; λ > 300 nm, 25 °C). Photoctivity after centrifugation, washing, re-centrifugation and re-suspension in fresh EDTA (0.1 M, pH 4.5) is also shown.

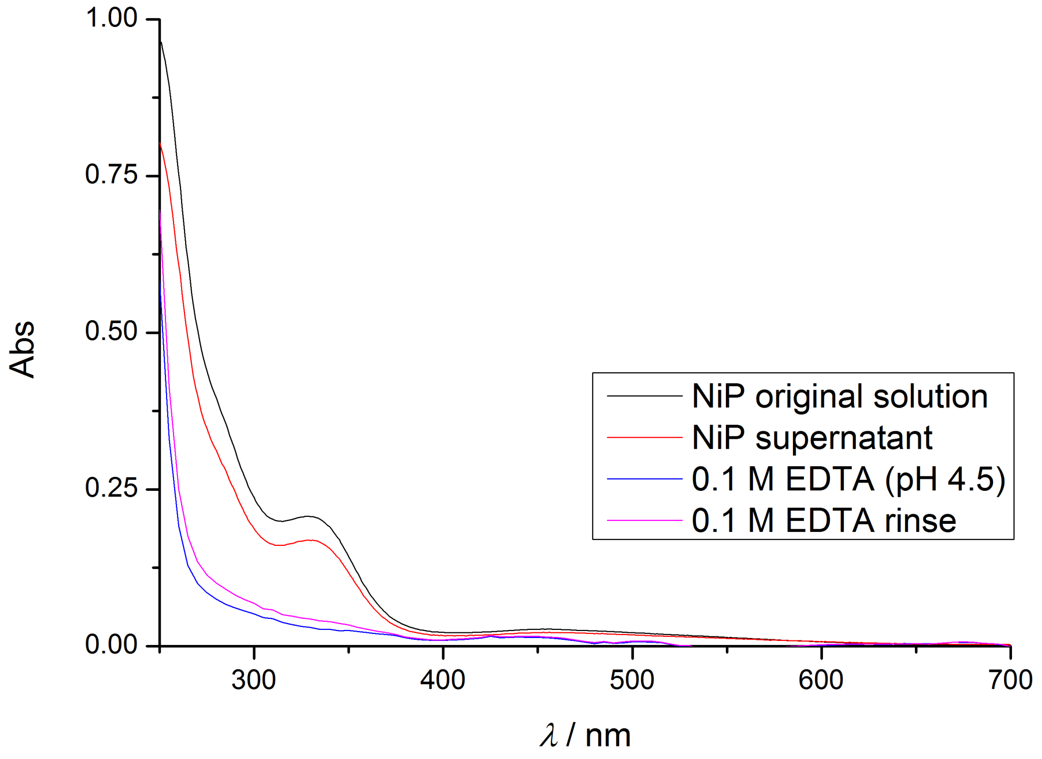

Figure S19. UV-Visible spectrum of NiP (20 nmol in 3 mL) in solution and again after addition of CN_x followed by centrifugation and measuring a UV-vis spectrum of the NiP remaining in the supernatant.

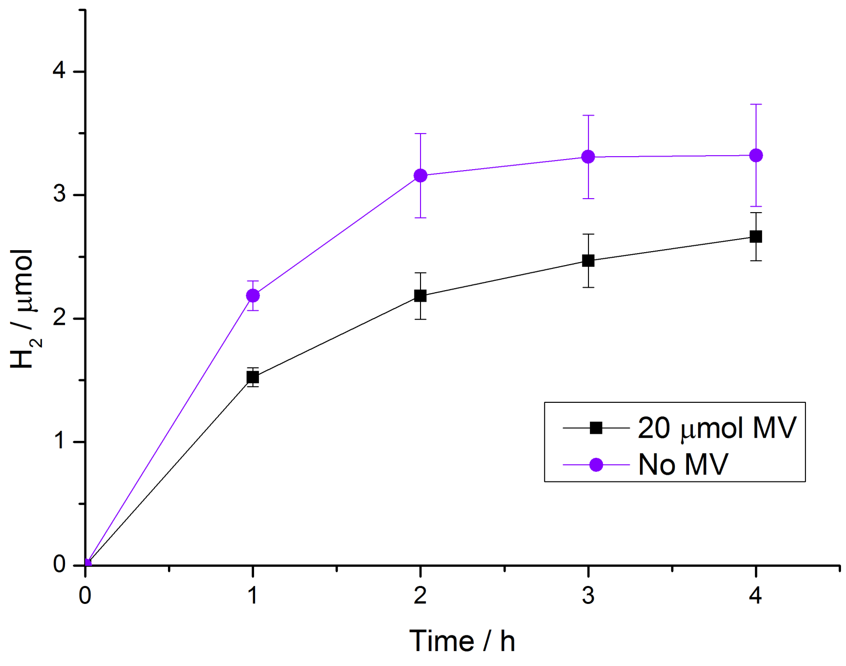

Figure S20. Hydrogen production with NiP (20 nmol) with CN_x (5 mg) in aqueous EDTA solution (0.1 M, pH 4.5) under 1 sun irradiation (100 mW cm^{-2}, λ > 300 nm, 25 °C) in the presence and absence of MV (20 µmol).

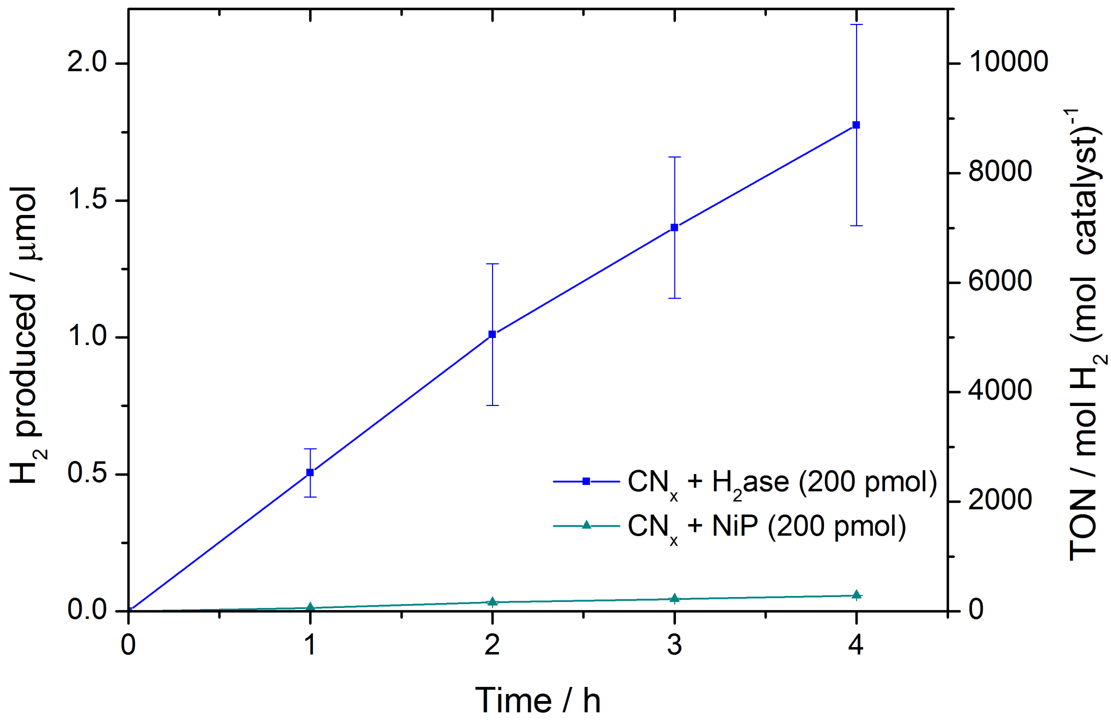

Figure S21. Hydrogen production and TON with NiP (200 pmol) with CN_x (5 mg) in aqueous EDTA solution (0.1 M, pH 4.5) and H_2ase (200 pmol) with CN_x (5 mg) in aqueous EDTA solution (0.1 M, pH 6) under 1 sun irradiation (100 mW cm^{-2}, $\lambda > 300$ nm, 25 °C).

End of Supporting Information
